# Extracellular vesicles-coupled miRNAs from oviduct and uterus modulate signaling pathways related to lipid metabolism and bovine early embryo development

**DOI:** 10.1186/s40104-024-01008-5

**Published:** 2024-04-04

**Authors:** Rosane Mazzarella, Karina Cañón-Beltrán, Yulia N. Cajas, Meriem Hamdi, Encina M. González, Juliano C. da Silveira, Claudia L. V. Leal, D. Rizos

**Affiliations:** 1 Department of Animal Reproduction, INIA-CSIC, Madrid, Spain; 2 Department of Biochemistry and Molecular Biology, Veterinary Faculty, Complutense University of Madrid (UCM), Madrid, Spain; 3 Department Agrarian Production, Technical University of Madrid, UPM, Madrid, Spain; 4 Departamento de Ciencias Biológicas, Universidad Técnica Particular de Loja,, UTPL, Loja, Ecuador; 5 Department of Anatomy and Embryology, FV-UCM, Madrid, Spain; 6 Department of Veterinary Medicine, FZEA-USP, Pirassununga, Brazil

**Keywords:** Embryo-maternal interaction, Epigenetic, Exosomes, Mammalian, Preimplantation, Reproductive fluids

## Abstract

**Background:**

Extracellular vesicles (EVs) present in oviductal (OF) and uterine fluid (UF) have been shown to enhance bovine embryo quality during in vitro culture by reducing lipid contents and modulating lipid metabolism-related genes (LMGs), while also influencing cell proliferation, suggesting their involvement on the regulation of different biological pathways. The regulation of signaling pathways related to cell differentiation, proliferation, and metabolism is crucial for early embryo development and can determine the success or failure of the pregnancy. Bioactive molecules within EVs in maternal reproductive fluids, such as microRNAs (miRNAs), may contribute to this regulatory process as they modulate gene expression through post-transcriptional mechanisms.

**Results:**

This study evaluated miRNA cargo in OF-EVs from the early luteal phase and UF-EVs from the mid-luteal phase, coinciding with embryo transit within oviduct and uterus in vivo, and its possible influence on LMGs and signaling pathways crucial for early embryo development. A total of 333 miRNAs were detected, with 11 exclusive to OF, 59 to UF, and 263 were common between both groups. From the 20 differentially expressed miRNAs, 19 up-regulated in UF-EVs (bta-miR-134, bta-miR-151-3p, bta-miR-155, bta-miR-188, bta-miR-181b, bta-miR-181d, bta-miR-224, bta-miR-23b-3p, bta-miR-24-3p, bta-miR-27a-3p, bta-miR-29a, bta-miR-324, bta-miR-326, bta-miR-345-3p, bta-miR-410, bta-miR-652, bta-miR-677, bta-miR-873 and bta-miR-708) and one (bta-miR-148b) in OF-EVs. These miRNAs were predicted to modulate several pathways such as Wnt, Hippo, MAPK, and lipid metabolism and degradation. Differences in miRNAs found in OF-EVs from the early luteal phase and UF-EVs from mid-luteal phase may reflect different environments to meet the changing needs of the embryo. Additionally, miRNAs may be involved, particularly in the uterus, in the regulation of embryo lipid metabolism, immune system, and implantation.

**Conclusions:**

Our study suggests that miRNAs within OF- and UF-EVs could modulate bovine embryo development and quality, providing insights into the intricate maternal-embryonic communication that might be involved in modulating lipid metabolism, immune response, and implantation during early pregnancy.

**Supplementary Information:**

The online version contains supplementary material available at 10.1186/s40104-024-01008-5.

## Introduction

Assisted reproduction techniques offer an alternative to enhance reproductive efficiency and address reproductive challenges in domestic species and humans. In bovine, in vitro embryo production has been widely employed in animal breeding programs to increase reproductive efficiency and genetic gain, serving both commercial and scientific purposes [1]. Despite its benefits, there is still a need for improvement, especially in embryo quality compared to the in vivo produced ones [2–4]. In vitro-produced embryos exhibit altered gene expression [4, 5], changed metabolism [6], lower cryotolerance [7], and lower pregnancy rates [8], characteristics that together explain their inferior quality when compared to their in vivo counterparts. Furthermore, factors such as higher lipid contents in embryos produced in vitro have been associated with lower quality and cryotolerance [3, 9]. One of the most critical obstacles in embryo production involves establishing an in vitro environment that replicates the physiological conditions as efficiently as possible.

In vivo, gametes and embryos undergo a series of physiological processes for successful reproduction. Together, these events support the adequate competence of oocytes and sperm for fertilization and subsequent embryo development [10]. In contrast, in vitro production occurs within a controlled and static environment. Therefore, one of the strategies used to improve embryonic development in vitro involves replicating, even if partially, the physiological conditions observed in vivo. Early embryo development initiates within the oviduct, where the embryo remains in the early luteal phase of the estrous cycle for approximately four days before it enters the uterus during the mid-luteal phase [11]. Throughout this period, the embryo develops mainly supported by the nutrients present in the oviductal fluid (OF) and uterine fluid (UF) until it undergoes implantation around the 19^th^–20^th^ day of pregnancy [12]. For this reason, it is imperative to investigate the physiological processes taking place in the maternal reproductive tract, with particular emphasis on the oviductal and uterine environments, to enhance the in vitro environment and better support preimplantation embryonic development in vitro.

Previous studies have shown that supplementing the in vitro culture (IVC) medium with OF and UF favors early embryonic development and improves bovine blastocyst quality [13]. Hamdi et al. [13] observed that this supplementation favors embryo methylation and enhances antioxidant activity. Moreover, the inclusion of OF during IVC increases trophectoderm cells’ number and modifies the expression of important developmental genes [14]. Additionally, embryos submitted to IVC in a medium conditioned by bovine oviduct epithelial cells (BOECs) have shown improved quality and cryotolerance [15]. These improvements are potentially mediated, at least in part, by the presence of EVs found in both OF and UF, which are absent in usual in vitro culture systems.

Extracellular vesicles have been identified as important components of bovine OF [14, 16, 17] and UF [18]. When supplemented in an IVC medium, EVs isolated either from BOECs conditioned medium [15], OF [14, 19] or UF [20], were found to be internalized and favored the development and quality of bovine embryos. Recently, sequential use of EVs from OF and UF during bovine embryo IVC also improved embryo quality by lowering lipid contents [21]. These effects were attributed, at least in part, to the modulation of gene expression related to lipid metabolism, such as CD36, PPARGC1B, FASN, PLIN2, and PNPLA2. Moreover, effects on cell proliferation, as observed by increased cell numbers in blastocysts cultured with EVs, were also detected, suggesting that EVs may modulate other cell signaling pathways beyond those related to lipid metabolism [21].

Extracellular vesicles carry bioactive molecules, including proteins, lipids, mRNAs, and noncoding RNAs, such as microRNAs (miRNAs) [22]. As reviewed by Gebert and MacRae [23] miRNAs are small noncoding molecules (∼22 nucleotides long) that function in the post-transcriptional regulation [24], and can be selectively loaded into EVs with the help of RNA-binding proteins [25, 26]. Furthermore, during early embryo development, miRNA modulation plays a role in maternal transcripts’ degradation [27], activation of the embryonic genome [28], embryo development [29], embryonic stem cell differentiation into trophectoderm cells [30], embryo hatching [31], embryo implantation competency [32], and pregnancy establishment [33]. Studies also suggest that miRNAs are involved in the maternal-embryo dialogue and dynamic changes during the pre-implantation to receptive phases [34], modulating signaling pathways essential for early embryo development and viability.

In summary, EVs have been studied in the female reproductive fluids, including bovine oviductal and uterine fluids. These EVs contain miRNAs, which are believed to play an important role in modulating embryo-maternal communication during early embryo development. EVs can mediate the delivery of this miRNA content to embryos, thereby influencing embryonic development and maternal-embryo communication. Furthermore, Almiñana et al. [19] identified several mRNAs and miRNAs related to lipid metabolism in oviductal EVs. These miRNAs could potentially impact the expression of lipid metabolism-related genes (LMGs) in embryos exposed to such EVs. In a previous study, we also detected miRNAs in the OF- and UF-EVs, which were utilized during embryo culture [21]. To further explore the role of miRNAs carried by EVs, the objective of this work was to investigate their potential involvement in regulating transcripts associated with lipid metabolism and other essential functions crucial for embryo development. This investigation was accomplished through rigorous bioinformatics analyses.

## Materials and methods

Unless otherwise stated, all chemicals utilized in this study were purchased from Merck KGaA, Darmstadt, Germany.

### Source of miRNA data used in this study

#### EVs isolation and characterization

The miRNA data employed in this study were obtained from EVs that were isolated and characterized as described by Cañón-Beltrán et al. [35]. Briefly, bovine oviducts and uteri from slaughtered heifers were selected according to the stage of the corpus luteum. Three oviducts corresponding to Stage 1 (from d 1 to 4 of the estrous cycle) and 3 uteri to Stage 2 (from d 5 to 10), according to Ireland et al. [36], were used. Additionally, only oviducts and uteri located on the same side (ipsilateral) of the corpus luteum were used in this study. The oviducts and uteri were flushed with calcium and magnesium-free phosphate-buffered saline (PBS^−^) and EVs were isolated from these flushing using size exclusion chromatography followed by ultracentrifugation for EVs concentration. EVs were characterized by Western blotting to detect EVs-marker proteins (CD9, HSP70, ALIX and CANX), nanoparticle tracking analysis for particle size and concentration determination, and transmission electron microscopy for morphology assessment. Part of the EVs were used in embryo in vitro culture experiments (oviduct EVs from d 1 to 4 and uterine EVs from d 5 to 8). Another portion of the EVs was dedicated to miRNA content analyses, as documented in Leal et al. [21].

#### miRNA contents analysis

miRNA contents were analyzed as described by Da Silveira et al. [37]. Total RNA (including small RNAs) was extracted from OF-EV and UF-EV samples (*n* = 3/group) using the miRNeasy Mini kit (QIAGEN; 217004) according to the manufacturer’s instructions. Reverse transcription was conducted on the total RNA (120 ng/sample), using the miScript PCR System, following the manufacturer’s instructions (QIAGEN, 218161). Briefly, total RNA, including the small RNA fraction, was incubated with 5 × miScript Hiflex Buffer, 10 × miScript Nucleic mix, RNase-free water, and miScript reverse transcriptase at 37 °C for 60 min, followed by 5 min at 95 °C.

The relative abundance levels of 382 mature miRNAs were determined using quantitative real-time PCR (qRT-PCR) in a 384-well plate, using the miScript SYBR Green PCR kit (QIAGEN, 218073) and the QuantStudio 6 Real-Time PCR System (Applied Biosystems). Briefly, a master mix in 6-μL reactions containing 2 × QuantiTect SYBR Green PCR Master Mix, 10 × miScript Universal Primer, miRNA-specific forward primer, and a 0.024-μL of 1:4 diluted cDNA was prepared. The PCR cycle conditions were 95 °C for 15 min, 45 cycles of 94 °C for 10 s, 55 °C for 30 s, and 70 °C for 30 s followed by a melt curve analysis to confirm the amplification of cDNA products.

To quantify miRNA expression levels, raw cycle threshold (Ct) values were normalized using the geometric mean of bta-miR-99b, Hm/Ms/Rt T1 snRNA, and RNT43 snoRNA, as internal controls. Three samples of OF-EVs and three samples of UF-EVs were utilized in the study. Only miRNAs with a Ct value less than 37 and detected in at least 2 out of the 3 samples in each group (OF- and UF-EVs), were considered to be present. A preliminary analysis was conducted, and 333 mature miRNAs were detected in both sample types (OF- and UF-EVs). Among these miRNAs, 20 exhibited differential expression. Specifically, 19 miRNAs were up-regulated in UF-EVs, while one miRNA showed upregulation in OF-EVs, as reported in the study by Leal et al. [21].

#### Statistical analysis

Statistical analyses were conducted using SAS 9.3 Software (SAS Institute). Normality was verified using a Shapiro–Wilk test. Once the normality was confirmed, a Student’s *t*-test was used to assess statistical differences of the qRT-PCR. The relative expression values were calculated using the Ct method. Ct values were normalized using the geometric mean of internal controls (bta-miR-99b, Hm/Ms/Rt T1 snRNA, and RNT43 snoRNA) and transformed by 2^−∆Ct^ for graphical representation of the relative transcript levels. A significance level of 5% was considered to determine statistical significance. In the graphical representation of miRNA data, the mean ± SEM (standard error of the mean) was used.

### Bioinformatics analyses of miRNAs

To gain deeper insights into the roles of detected miRNAs and their possible connection with the effects of EVs added to the bovine IVC media, in this study, the miRNA dataset was submitted to bioinformatics analyses to identify the pathways that are being modulated by OF- and UF-miRNAs.

The chromosome location of differentially detected miRNAs was accessed through the RNAcentral v20 tool (https://rnacentral.org). This tool imports genome locations from Ensembl, miRBase, and others databases, and maps the sequences to the reference genomes downloaded from Ensembl and Ensembl Genomes using blat. The structure of miRNAs precursor family and sequence conservation across species of miRNA precursor family were accessed through Rfam 14.8 database (https://rfam.org) or miRNAs precursor secondary structure generated by R2DT 1.2 software (RNA 2D Templates) using template provided by Rfam.

miRNAs differently detected between groups and those exclusive to each group were loaded within the miRWalk 3.0 database (http://mirwalk.umm.uni-heidelberg.de/search_mirnas). Based on the gene target with prediction score ≥ 0.95, the program generates a list of possible pathways regulated by the selected miRNAs. The list of predicted pathways is based on the number of targeted genes predicted to be regulated by the number of the selected miRNAs generating a *P*-value for the interaction between miRNAs and their targeted genes. Only pathways with a *P*-value lower than 0.05 were considered as significant. Pathways classification according to its biological function were accessed from the Kyoto Encyclopedia of Genes and Genomes (KEGG, https://www.genome.jp/kegg). Venn’s diagrams for comparing lists of miRNAs and biological pathways were drawn using the online tool Venny 2.1.0 BioinfoGP (https://bioinfogp.cnb.csic.es/tools/venny/index.html).

miRBase (http://www.mirbase.org) was used in order to identify the sequence similarities between bovine miRNA and human sequences for downstream analysis. Only sequences with 90%–100% of similarity and with preserved seed region were used.

Enriched terms identification, Gene Ontology (GO) analysis, and protein–protein interaction enrichment analysis were performed with Metascape (https://metascape.org) with default parameters. Initially, two different databases were used to identify miRNA-mRNA target interactions: miRTarBase (https://mirtarbase.cuhk.edu.cn) and TarBase v.8 (https://dianalab.e-ce.uth.gr/html/diana/web/index.php?r=tarbasev8%2Findex). Venn diagrams were used for comparing the list of genes and to identify genes regulated by miRNAs differently expressed in OF-EVs and UF-EVs present in both databases. The resulting gene list was uploaded onto Metascape, which generates, for each given gene list, pathway and process enrichment analysis with the following ontology sources: KEGG Pathway, GO Biological Processes, Reactome Gene Sets, Canonical Pathways, CORUM, WikiPathways, and PANTHER Pathway. All genes in the human genome are used as the enrichment background and terms with a *P*-value < 0.01, a minimum count of 3, and an enrichment factor > 1.5 are collected and grouped into clusters based on their membership similarities. The most statistically significant term within a cluster is chosen to represent the cluster. The networks are visualized using Cytoscape. For protein–protein interaction enrichment analysis, Metascape analysis is carried out with the following databases: STRING6, BioGrid7, OmniPath8, and InWeb_IM9. Only physical interactions in STRING (physical score > 0.132) and BioGrid are used.

As miRNA targeted genes to investigate, we have selected LMGs based on our previous study that showed that in bovine embryos cultured in vitro in the presence of EVs from OF and UF, lipid contents were reduced and some LMGs had altered expression [21]. Selected LMGs are related to different processes of lipid metabolism, including lipid uptake (*LDLR* and *CD36*), lipid transport (*FABP3*), lipid accumulation (*PLIN2*), lipogenesis (*PPARGC1B*, *ACACA*, and *FASN*), and lipolysis (*PNPLA2* and *LIPE*). Fatty acid synthesis genes also previously described by Sudano et al. [38] as expressed by bovine embryos in another study were additionally analyzed (ACSL3, ELOV5, and ELOV6). These interactions were studied using the QIAGEN Ingenuity Pathway Analysis software, (QIAGEN Inc., https://www.qiagenbioinformatics.com/products/ingenuitypathway-analysis). Differentially expressed miRNAs were used as an input and only human sequences with 90%–100% of similarity with the bovine and with preserved seed region were used. IPA allowed the data to be interrogated to determine the functions associated with each miRNA and to identify which target gene from a given list interacted with the differentially expressed miRNA. The list of investigated LMGs is provided below, and the selected functions of interest included "uptake of lipid", "transport of lipid", "synthesis of lipid", "accumulation of lipid droplets", and "lipolysis". Interaction networks were constructed by identifying genes targeted by differentially expressed miRNAs, creating networks with a maximum of 25 molecules for each function. All networks exclusively incorporated experimentally observed relationships, excluding predicted bindings.

## Results

### miRNA in EVs from OF and UF

**Fig. 1:**
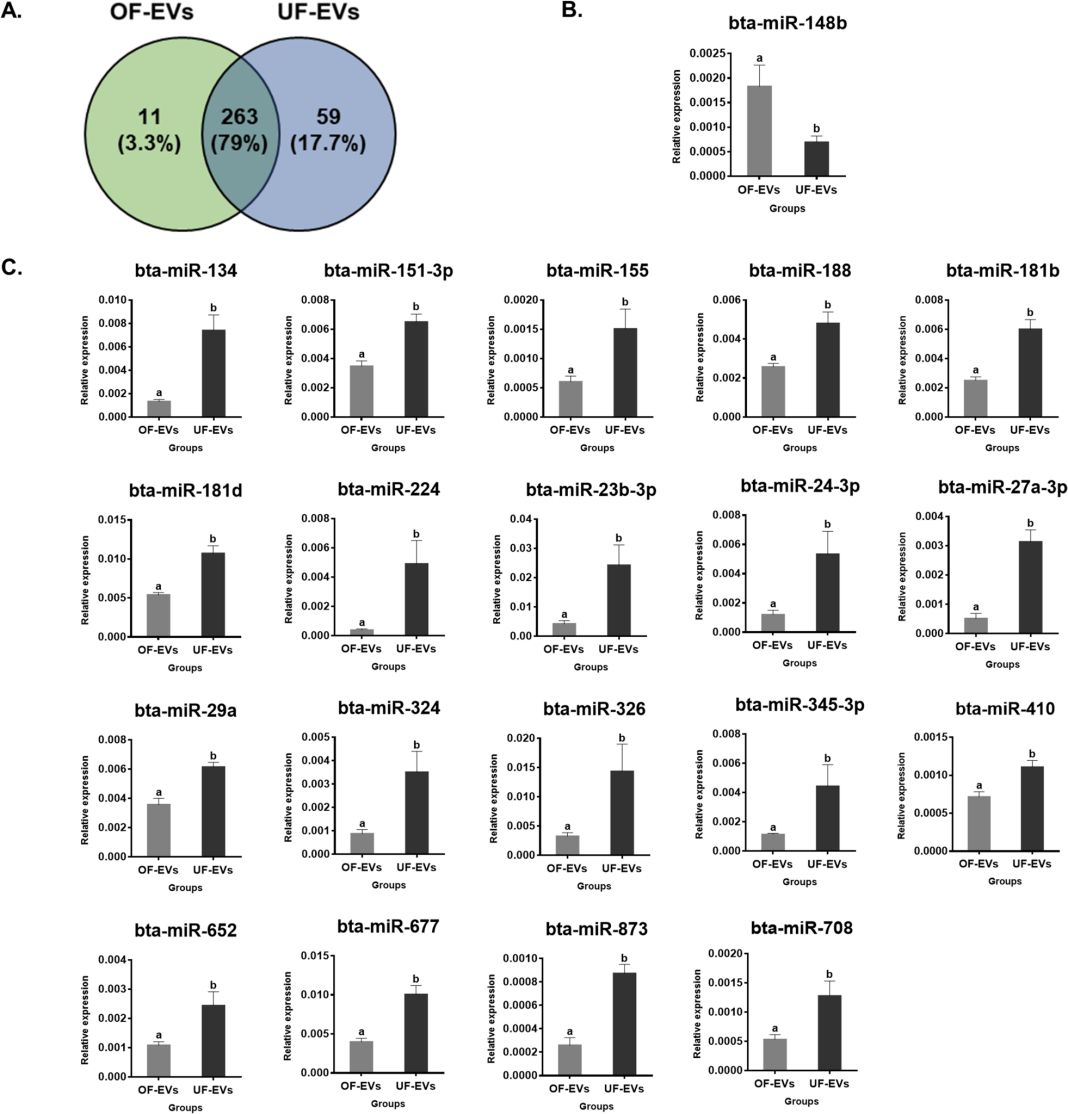
miRNAs profile of OF-EVs and UF-EVs.** A** Venn diagram representing the 333 miRNAs detected: 263 in common between the two groups, 11 exclusives to OF-EVs, and 59 exclusive to UF-EVs. **B** miRNA (bta-miR-148b) up-regulated in OF-EVs. **C** miRNAs (bta-miR-134, bta-miR-151-3p, bta-miR-155, bta-miR-188, bta-miR-181b, bta-miR-181d, bta-miR-224, bta-miR-23b-3p, bta-miR-24-3p, bta-miR-27a-3p, bta-miR-29a, bta-miR-324, bta-miR-326, bta-miR-345-3p, bta-miR-410, bta-miR-652, bta-miR-677, bta-miR-873 and bta-miR-708) up-regulated in UF-EVs. ^a,b^Different letters indicate miRNAs with relative expression significantly different (*P* < 0.05) between the groups. Error bars represent SEM

Previously, 333 mature miRNAs had been detected in both sample types, and 20 of them were differentially expressed [21]. The present analyses of the data set revealed that from these 333 miRNAs, 263 were common to both EVs types, 11 were exclusive for OF-EVs and 59 for UF-EVs, representing 79%, 3.3%, and 17.7%, respectively, of the total miRNAs identified (Fig. 1A). Among the 263 miRNAs detected in both groups, the 20 miRNAs differentially expressed (*P* < 0.05) between the groups are depicted in Fig. 1: one miRNA (bta-miR-148b) was up-regulated in OF-EVs (Fig. 1B) and 19 miRNAs (bta-miR-134, bta-miR-151-3p, bta-miR-155, bta-miR-188, bta-miR-181b, bta-miR-181d, bta-miR-224, bta-miR-23b-3p, bta-miR-24-3p, bta-miR-27a-3p, bta-miR-29a, bta-miR-324, bta-miR-326, bta-miR-345-3p, bta-miR-410, bta-miR-652, bta-miR-677, bta-miR-873 and bta-miR-708) were up-regulated in UF-EVs (Fig. 1C). Table 1 shows the miRNAs identified as exclusively detected in OF-EVs and UF-EVs.

**Table 1:** Exclusively detected miRNAs^1^

OF-EVs 11 exclusive miRNAs	UF-EVs 59 exclusive miRNAs				
bta-let-7a-5p	bta-miR-105a	bta-miR-193a	bta-miR-23b-5p	bta-miR-379	bta-miR-544b
bta-miR-18a	bta-miR-107	bta-miR-194	bta-miR-24	bta-miR-380-3p	bta-miR-551a
bta-miR-19a	bta-miR-101	bta-miR-196a	bta-miR-28	bta-miR-412	bta-miR-551b
bta-miR-218	bta-miR-133b	bta-miR-196b	bta-miR-302a	bta-miR-451	bta-miR-562
bta-miR-365-3p	bta-miR-133c	bta-miR-199a-3p	bta-miR-302c	bta-miR-448	bta-miR-758
bta-miR-376a	bta-miR-128	bta-miR-199a-5p	bta-miR-302d	bta-miR-455-5p	bta-miR-875
bta-miR-376e	bta-miR-136	bta-miR-199b	bta-miR-3064	bta-miR-499	bta-miR-95
bta-miR-539	bta-miR-140	bta-miR-208b	bta-miR-29b	bta-miR-487a	bta-miR-96
bta-miR-628	bta-miR-147	bta-miR-19b	bta-miR-329b	bta-miR-495	bta-miR-1298
bta-miR-876	bta-miR-150	bta-miR-212	bta-miR-33a	bta-miR-542-5p	bta-miR-1271
bta-miR-119	bta-miR-18b	bta-miR-215	bta-miR-369-5p	bta-miR-543	bta-miR-1282
	bta-miR-183	bta-miR-223	bta-miR-411c-3p	bta-miR-544a	

^1^Uniquely identified miRNAs include 11 exclusive to OF-EVs and 59 exclusive to UF-EVs

Upon analyzing the fold change of differentially expressed miRNAs between OF-EVs and UF-EVs (Table 2), several miRNAs exhibited the highest fold change (> 2.0), including bta-miR-27a-3p, bta-miR-23b-3p, bta-miR-134, bta-miR-24-3p, bta-miR-326, bta-miR-224 and bta-miR-324. Fold changes were calculated as the ratio of the mean expression level of UF-EVs/OF-EVs. Therefore, if the fold change is positive, it means that the miRNA is up-regulated in UF-EVs and down-regulated in OF-EVs; if the fold change is negative, it means it is down-regulated in UF-EVs and up-regulated in OF-EVs.

**Table 2:** Relative level of differentially expressed miRNAs in OF-EVs and UF-EVs (*n* = 3)^1^

microRNA	OF-EVs (Mean ± SEM)	UF-EVs (Mean ± SEM)	*P*-value	log_2_(Fold change)
bta-miR-148b	0.0018 ± 0.0004	0.0007 ± 0.0001	≤ 0.038	–1.4065
bta-miR-134	0.0013 ± 0.0002	0.0074 ± 0.0013	≤ 0.001	2.4870
bta-miR-151-3p	0.0035 ± 0.0004	0.0065 ± 0.0005	≤ 0.012	0.9047
bta-miR-155	0.0006 ± 0.0001	0.0015 ± 0.0003	≤ 0.037	1.3253
bta-miR-188	0.0026 ± 0.0002	0.0048 ± 0.0006	≤ 0.024	0.9014
bta-miR-181b	0.0025 ± 0.0003	0.0060 ± 0.0007	≤ 0.004	1.2652
bta-miR-181d	0.0054 ± 0.0003	0.0107 ± 0.0010	≤ 0.005	0.9930
bta-miR-224	0.0004 ± 0.0001	0.0052 ± 0.0019	≤ 0.005	3.7966
bta-miR-23b-3p	0.0042 ± 0.0012	0.0242 ± 0.0070	≤ 0.022	2.5391
bta-miR-24-3p	0.0012 ± 0.0003	0.0053 ± 0.0016	≤ 0.029	2.1562
bta-miR-27a-3p	0.0005 ± 0.0002	0.0031 ± 0.0004	≤ 0.030	2.6102
bta-miR-29a	0.0036 ± 0.0004	0.0061 ± 0.0003	≤ 0.038	0.7838
bta-miR-324	0.0009 ± 0.0002	0.0035 ± 0.0009	≤ 0.029	2.0001
bta-miR-326	0.0032 ± 0.0006	0.0143 ± 0.0047	≤ 0.046	2.1404
bta-miR-345-3p	0.0011 ± 0.0001	0.0044 ± 0.0015	≤ 0.048	1.9707
bta-miR-410	0.0007 ± 0.0001	0.0011 ± 0.0001	≤ 0.028	0.6311
bta-miR-652	0.0011 ± 0.0001	0.0024 ± 0.0005	≤ 0.042	1.1760
bta-miR-677	0.0040 ± 0.0005	0.0100 ± 0.0012	≤ 0.005	1.3403
bta-miR-873	0.0003 ± 0.0001	0.0009 ± 0.0001	≤ 0.021	1.7507
bta-miR-708	0.0005 ± 0.0001	0.0013 ± 0.0003	≤ 0.024	1.2640

^1^Fold changes were calculated as the ratio of the mean expression level of UF-EVs/OF-EVs

We also accessed the chromosome location, structure of miRNAs precursor family, and conservation of miRNA precursor family across species (Additional file 1). Domestic cattle (*Bos taurus*) have 60 chromosomes, including 58 autosomes and 2 sex chromosomes [39]. The chromosome locations were accessed through RNAcentral v20 tool and the differentially expressed miRNAs are in chromosomes 1, 4, 5, 7, 8, 11, 14, 15, 19, 21, and 29. The miRNAs bta-miR-188, bta-miR-224, and bta-miR-652 are located in the X sex chromosome. Conservation of miRNA precursor family was accessed through Rfam 14.8 database and showed that the precursor miRNAs of bta-miR-148b, bta-miR-134, bta-miR-155, bta-miR-224, bta-miR-24-3p and bta-miR-27a-3p have many nucleotides in common among mammals. The structure of miRNAs precursor family of bta-miR-188, bta-miR-181b, bta-miR-29a, bta-miR-326, bta-miR-410 and bta-miR-873 was generated through R2DT 1.2 software. There is no information available on family precursor structure and sequence conservation for bta-miR-151-3p, bta-miR-181d, bta-miR-23b-3p, bta-miR-324, bta-miR-345-3p, bta-miR-652, bta-miR-677 and bta-miR-708.

### Enrichment analysis of bovine miRNAs from OF-EVs and UF-EVs

To investigate the biological functions of differently expressed and exclusive miRNAs in OF-EVs and UF-EVs, bioinformatics analysis using bovine miRNAs was performed with miRWalk 3.0 database. The 11 miRNAs exclusive to OF-EVs are predicted to modulate 48 signaling pathways (Additional file 2A), and the 59 ones exclusive to UF-EVs are predicted to modulate 78 signaling pathways (Additional file 2B). miRNAs up-regulated in OF-EVs are predicted to modulate 17 signaling pathways (Additional file 2C), while those 19 miRNAs up-regulated in UF-EVs are predicted to modulate 101 signaling pathways (Additional file 2D).

**Fig. 2:**
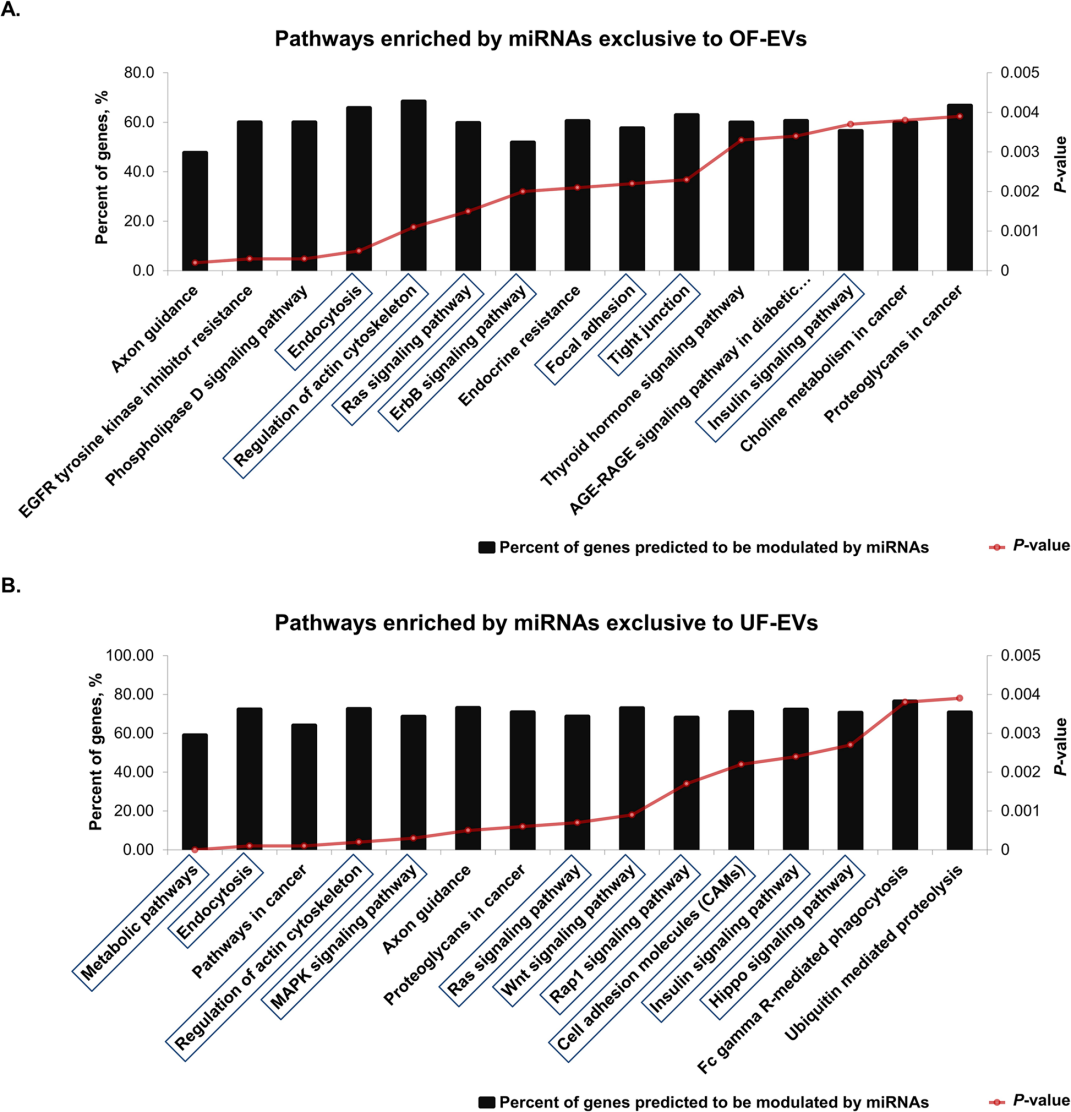
Biological pathways predicted as regulated by miRNAs exclusive to OF-EVs and UF-EVs. **A** Pathways predicted as regulated by miRNAs exclusive to OF-EVs. **B** Pathways predicted as regulated by miRNAs exclusive UF-EVs. The left *Y*-axis values represent the percentage of genes predicted as modulated by the miRNAs for the respective pathways (number of genes predicted to be modulated by miRNAs in each group divided by the total number of genes of each pathway). The right *Y*-axis represents the enrichment score (*P*-value) for each interaction. Blue squares highlight functions with potential relevance for embryo development. Pathways were accessed through miRWalk 3.0 database

Considering only the 15 signaling pathways with the most significant *P*-values in each group as mainly modulated by those miRNAs, the ones exclusively found in OF-EVs are predicted to modulate 2 pathways related with cancer, endocrine system (insulin signaling pathway), cellular processes (endocytosis, focal adhesion, and tight junctions) and signaling processes (RAS and ErbB) that can regulate cell proliferation, differentiation, cell motility, and survival (Fig. 2A). Exclusive miRNAs in UF-EVs are predicted to modulate 2 pathways related with cancer, metabolic pathways and pathways related with endocrine system (insulin signaling pathway), cellular processes (endocytosis, regulation of actin cytoskeleton and cell adhesion molecules) and signaling processes (MAPK, RAS, Wnt, Rap1 and Hippo) that modulate cell apoptosis, cell proliferation, differentiation, migration, among other cellular functions (Fig. 2B).

**Fig. 3:**
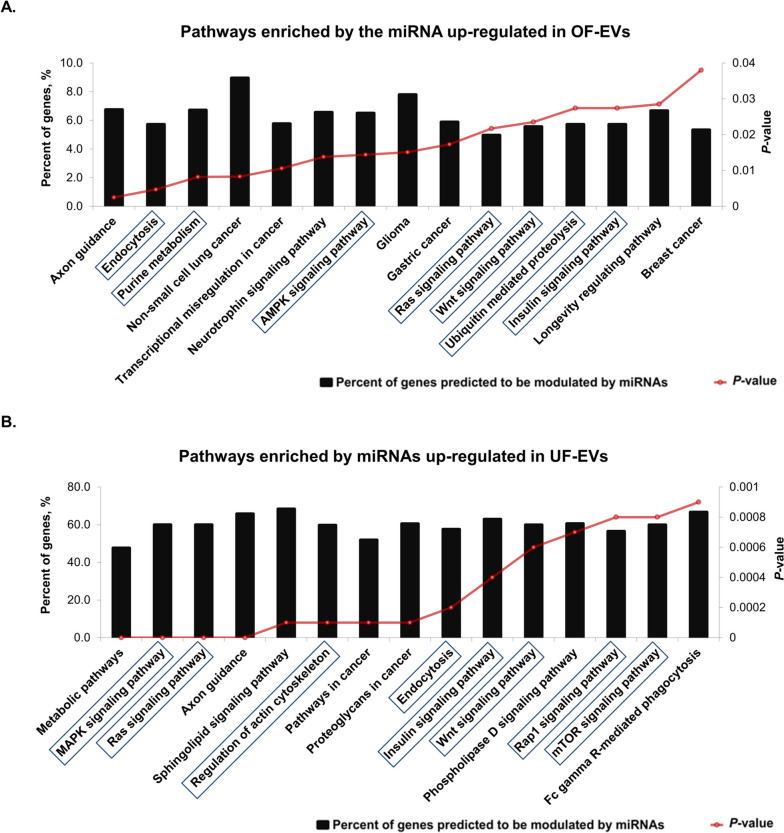
Biological pathways predicted as regulated by miRNAs up-regulated in OF-EVs and UF-EVs. **A** Pathways predicted as regulated by miRNA (bta-miR-148b) up-regulated in OF-EVs. **B** Pathways predicted as regulated by miRNAs (bta-miR-134, bta-miR-151-3p, bta-miR-155, bta-miR-188, bta-miR-181b, bta-miR-181d, bta-miR-224, bta-miR-23b-3p, bta-miR-24-3p, bta-miR-27a-3p, bta-miR-29a, bta-miR-324, bta-miR-326, bta-miR-345-3p, bta-miR-410, bta-miR-652, bta-miR-677, bta-miR-873 and bta-miR-708) up-regulated in UF-EVs. The left *Y*-axis values represent the percentage of genes predicted as modulated by the miRNAs for the respective pathways (number of genes predicted to be modulated by miRNAs in each group divided by the total number of genes of each pathway). The right *Y*-axis represents the enrichment score (*P*-value) for each interaction. Blue squares highlight functions with potential relevance for embryo development. Pathways were accessed through miRWalk 3.0 database

miRNAs up-regulated in OF-EVs are predicted to modulate 5 pathways related with cancer, metabolism (purine metabolism), cellular processes (endocytosis), endocrine system (insulin signaling pathway), and signaling processes (AMPK, Ras, and Wnt) that regulate cell cycle, proliferation, survival, growth, migration, and differentiation (Fig. 3A). Up-regulated in UF-EVs are predicted to modulate 2 pathways related with cancer, metabolic pathways, cellular processes (endocytosis and regulation of actin cytoskeleton), endocrine system (insulin signaling pathway) and signaling processes (MAPK, Ras, Wnt, Rap1 and mTOR) that control cell cycle, cell–cell junction formation and cell polarity, cytoskeletal organization, metabolism, and survival (Fig. 3B). Moreover, critical pathways involved in embryonic cell fate, proliferation, survival, and growth, such as RAS, Wnt, and endocytosis, are found among the 15 signaling pathways with the most significant *P*-values for both OF-EVs and UF-EVs. However, it is important to note that these pathways may undergo distinct modulation patterns, as different miRNA-mRNA interactions occur in the oviduct and the uterus (Table 3).

**Table 3:** Pathways regulated within the oviduct and uterus, and target genes of differentially expressed miRNAs on the OF- or UF-EVs^1^

**Pathways**	**Target genes**	**OF-EVs**		**UF-EVs**	
		**Exclusive miRNAs (11)**	**Up-regulated miRNA (1)**	**Exclusive miRNAs (59)**	**Up-regulated miRNAs (19)**
Endocytosis	Total	59 genes	14 genes	177 genes	141 genes
	Exclusively modulated	3 (*FAM21A*, *LOC616942*, *HSPA1A*)	1 (*AP2A2*)	42 (*CCR5*, *CAPZA2*, *ARPC1B*, *ARF3*, *VPS25*, *MVB12A*, *AP2M1*, *ARPC2*, *VPS28*, *VTA1*, *BOLA*, *RAB4A*, *HGS*, *SNX6*, *CAV3*, *SNF8*, *CHMP6*, *STAMBP*, *AP2S1*, *USP8*, *RAB5A*, *SNX4*, *TSG101*, *CBLC*, *RAB8A*, *AP2A1*, *EHD4*, *RAB11FIP2*, *VPS26B*, *PRKCI*, *TFRC*, *CLTB*, *CXCR4*, *GRK5*, *HSPA8*, *IGF2R*, *SPG21*, *PIP5K1B*, *WASHC5*, *GRK4*, *ARAP1*, *IZUMO1R*)	14 (*VPS29*, *RAB11B*, *CHMP2B*, *JSP1*, *FOLR2*, *CYTH2*, *SPART*, *SNX3*, *ACAP2*, *BoLA*, *PSD*, *FGFR4*, *ARRB2*, *FOLR3*)
Ras signaling	Total	56 genes	12 genes	0 genes	145 genes
	Exclusively modulated	1 (*PLA2G12B*)	1 (*KSR*)	30 (*PLA2G2D1*, *STK4*, *PLA2G4A*, *FGF18*, *PAK4*, *RAB5A*, *IGF1*, *RRAS2*, *RAC3*, *RASAL3*, *MAP2K1*, *RASGRP1*, *CALM*, *FGF20*, *RIN1*, *GNB3*, *PLA2G4F*, *FGF16*, *EXOC2*, *PAK2*, *CALM2*, *IKBKB*, *IKBKG*, *VEGFB*, *VEGFC*, *PIK3R2*, *LOC615045*, *PLA2G2C*, *LOC786717*, *LOC789148*)	14 (*PLA1A*, *PLA2G16*, *PRKCG*, *INS*, *FGFR4*, *TBK1*, *EPHA2*, *PIK3CB*, *GNG2*, *PLCG1*, *PRKCA*, *RASA1*, *LOC521224*, *SHC2*)
Insulin signaling	Total	8 genes	35 genes	101 genes	87 genes
	Exclusively modulated	1 (*PDPK*)	0	23 (*HK1*, *RHEB*, *PPP1CA*, *PKLR*, *PRKAR1A*, *EIF4EBP1*, *LIPE*, *PTPN1*, *PRKAA1*, *MAP2K1*, *CALM*, *INPP5J*, *ACACB*, *PPP1R3A*, *PRKCI*, *CALM2*, *IKBKB*, *PIK3R2*, *PCK1*, *MTOR*, *PHKA1*, *HK2*, *PRKAG2*)	9 (*FLOT1*, *INS*, *PHKA2*, *GYS2*, *RPS6KB2*, *PIK3CB*, *G6PC3*, *TSC2*, *SHC2*)
Wnt signaling	Total	0 genes	9 genes	118 genes	97 genes
	Exclusively modulated	0	0	30 (*WNT2*, *PRICKLE3*, *SFRP2*, *MYC*, *SFRP4*, *WIF1*, *CTNNB1*, *DAAM1*, *DKK2*, *PPARD*, *MAPK10*, *CSNK1B*, *RAC3*, *FZD1*, *PORCN*, *NFATC4*, *ROR2*, *PLCB4*, *RNF43*, *AXIN1*, *AXIN2*, *SIAH1*, *GPC4*, *PPP3R1*, *CSNK2A1*, *CSNK1A1*, *APC2*, *RYK*, *EP300*, *CTBP1*)	8 (*WNT16*, *CAMK2D*, *RSPO3*, *WNT10A*, *CREBBP*, *PRKCG*, *PRKCA*, *LOC780968*)
Regulation of actin cytoskeleton	Total	51 genes	0 genes	153 genes	126 genes
	Exclusively modulated	1 (*MYL2*)	0	30 (*ARPC1B*, *PFN1*, *GSN*, *ARPC2*, *PPP1CA*, *ACTN1*, *FGF18*, *PAK4*, *PFN3*, *MYLK2*, *RRAS2*, *RAC3*, *ITGB7*, *MAP2K1*, *FGF20*, *DIAPH3*, *FGF16*, *CYFIP1*, *PAK2*, *LPAR1*, *CXCR4*, *ITGAV*, *ITGB1*, *PIK3R2*, *ITGA4*, *ITGB2*, *APC2*, *PIP5K1B*, *ARHGEF1*, *PPP1R12C*)	4 (*INS*, *FGFR4*, *ITGB4*, *PIK3CB*)

^1^Although predicted to be modulated by both OF- and the UF-EVs, it is important to notice that biological pathways such as RAS, Wnt, and endocytosis may undergo distinct modulation patterns, since different miRNA-mRNA interactions are reported from miRWalk 3.0 platform

**Fig. 4:**
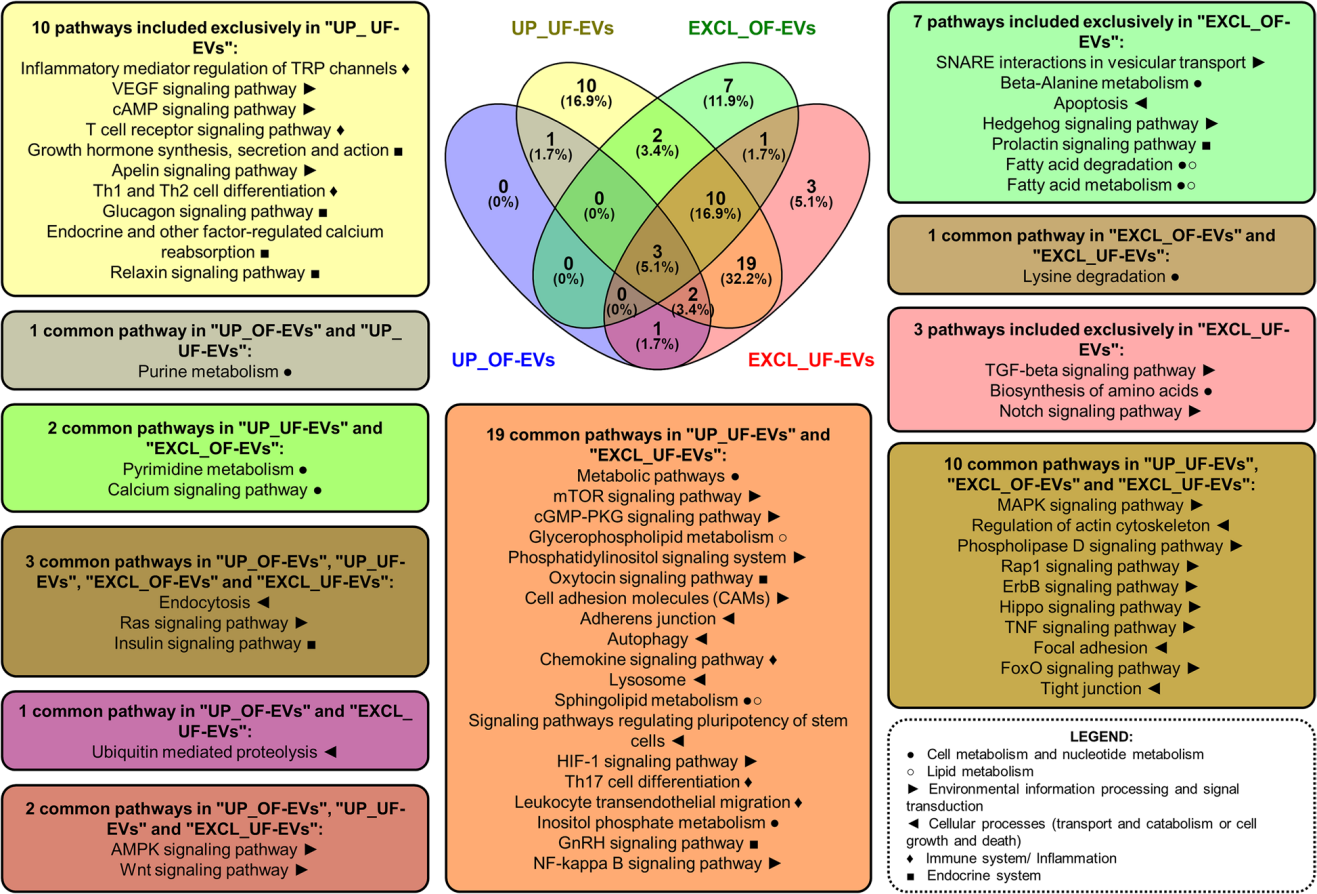
Venn diagram of pathways modulated by differently expressed and exclusive miRNAs in OF-EVs and UF-EVs. Pathways were selected based on their potential relevance for embryo development. Pathways classification according to its biological function were accessed from the Kyoto Encyclopedia of Genes and Genomes (KEGG). UP_OF-EVs: pathways predicted as modulated by miRNAs up-regulated in oviductal fluid extracellular vesicles. EXCL_OF-EVs: pathways predicted as modulated by miRNAs exclusive to oviductal fluid extracellular vesicles. UP_UF-EVs: pathways predicted as modulated by miRNAs up-regulated in uterine fluid extracellular vesicles. EXCL_UF-EVs: pathways predicted as modulated by miRNAs exclusive to uterine fluid extracellular vesicles

Additionally, a Venn diagram was created to illustrate the pathways relevant for embryo development that are predicted to be modulated by differentially expressed and exclusive miRNAs in OF-EVs and UF-EVs. The diagram aims to identify and isolate the pathways that are uniquely modulated within the uterus or the oviduct, as well as the pathways that are shared between them (Fig. 4). Pathways were classified according to their biological function using the KEGG. In the oviduct, no pathway was identified as regulated by the only miRNA up-regulated in OF-EVs. Seven pathways, representing 11.9% of all pathways, were identified as modulated only by miRNAs exclusive in OF-EVs, and 3 are related to cell metabolism, including 2 pathways related with lipid metabolism and degradation, and pathways related with embryo development, such as Hedgehog and apoptosis.

In the uterus, 3 pathways representing 5.1% of all identified pathways, are modulated only by miRNAs exclusive to UF-EVs: one related with metabolism (biosynthesis of amino acids) and 2 signaling pathways important for embryo development (TGF-β and Notch). Additionally, 10 pathways, representing 16.9% of all pathways, are modulated only by miRNAs up-regulated in UF-EVs and 4 are related with endocrine system (hormonal regulation and growth factors), 3 with immune system and inflammation, and 3 with signal transduction such as Apelin signaling pathway (regulating apoptosis and proliferation).

The largest group of elements (19 pathways, 32.2% of all identified pathways) corresponds to pathways modulated by miRNAs in the uterus, either by miRNAs up-regulated in UF-EVs or by miRNAs exclusive to UF-EVs. Among those, 6 are related with signaling pathways (mTOR, cGMP/PKG, phosphatidylinositol, CAMs, HIF-1 e NFkB); 4 with cellular processes (adherens junction, autophagy, lysosome and importantly, signaling pathways regulating pluripotency of stem cells); 3 pathways are related with immune system and inflammation (chemokine, Th17 cell differentiation and leukocyte migration); 2 related with the endocrine system (oxytocin and GnRH signaling pathway); 2 with lipid membrane metabolism (such as glycerophospholipid metabolism); and pathways related with the endocrine system (GnRH).

Important pathways for embryo development (10 pathways, 16.9% of all selected pathways) are also modulated by miRNAs up-regulated in UF-EVs and also exclusive for OF- and UF-EVs. Most of those (7 pathways) are related with environmental information processing and signal transduction processes, such as MAPK, Rap1, ErbB, Hippo, TNF, and FoxO signaling pathways; all the remainder are related with cellular processes, including regulation of actin cytoskeleton, focal adhesion, and tight junction. Also, 2 important pathways for embryo development (AMPK and Wnt) are regulated by miRNAs up-regulated in OF-EVs and by miRNAs up-regulated in UF-EVs. Additionally, 3 pathways are commonly modulated between all the groups and are related with environmental information processing and signal transduction (Ras signaling pathway), cellular processes (endocytosis), and endocrine system (insulin signaling pathway).

### Gene Ontology and protein–protein interaction analysis of bovine miRNAs from OF-EVs and UF-EVs

To further investigate the role of miRNAs from OF-EVs and UF-EVs, Metascape tool was used for the identification of enriched terms, GO analysis, and protein–protein interaction enrichment analysis. Initially, similarities between bovine miRNA and human sequences for upstream analysis were assessed. Only sequences with 90%–100% similarity and with preserved seed region were used (Table 4). Next, 2 different databases were used to identify miRNA-mRNA target interactions (MiRTarBase and TarBase v.8), and Venn diagrams were used for comparing the list of genes and to identify genes regulated by miRNAs differently expressed in OF-EVs and UF-EVs present in both databases (Additional files 3 and 4). The genes in common between the two databases were used for upstream analyses.

**Table 4:** Sequence similarities between bovine miRNA and human sequences^1^

Bovine			Human			
miRNA ID	**Accession**	**Sequence ( 5'→3')**	**miRNA ID**	**Accession**	**Sequence ( 5'→3')**	**Similarity, %**
bta-miR-134	MIMAT0009227	U**GUGACUG**GUUGACCAGAGUGG	hsa-miR-134-5p	MIMAT0000447	U**GUGACUG**GUUGACCAGAGGGG	95.45
bta-miR-148b	MIMAT0003814	U**CAGUGCA**UCACAGAACUUUGU	hsa-miR-148b-3p	MIMAT0000759	U**CAGUGCA**UCACAGAACUUUGU	100.00
bta-miR-151-3p	MIMAT0003524	C**UAGACUG**AAGCUCCUUGAGG	hsa-miR-151a-3p	MIMAT0000757	C**UAGACUG**AAGCUCCUUGAGG	100
bta-miR-155	MIMAT0009241	U**UAAUGCU**AAUCGUGAUAGGGGU	hsa-miR-155-5p	MIMAT0000646	U**UAAUGCU**AAUCGUGAUAGGGGU	100
bta-miR-188	MIMAT0009249	C**AUCCCUU**GCAUGGUGGAGGGU	hsa-miR-188-5p	MIMAT0000457	C**AUCCCUU**GCAUGGUGGAGGG	95.45
bta-miR-181b	MIMAT0003793	A**ACAUUCA**UUGCUGUCGGUGGGUU	hsa-miR-181b-5p	MIMAT0000257	A**ACAUUCA**UUGCUGUCGGUGGGU	95.65
bta-miR-181d	MIMAT0009243	A**ACAUUCA**UUGUUGUCGGUGGGU	hsa-miR-181d-5p	MIMAT0002821	A**ACAUUCA**UUGUUGUCGGUGGGU	100
bta-miR-224	MIMAT0009271	**CAAGUCA**CUAGUGGUUCCGUUUA	hsa-miR-224-5p	MIMAT0000281	U**CAAGUCA**CUAGUGGUUCCGUUUAG	92
bta-miR-23b-3p	MIMAT0003852	A**UCACAUU**GCCAGGGAUUACCAC	hsa-miR-23b-3p	MIMAT0000418	A**UCACAUU**GCCAGGGAUUACCAC	100.00
bta-miR-24-3p	MIMAT0003840	U**GGCUCAG**UUCAGCAGGAACAG	hsa-miR-24-3p	MIMAT0000080	U**GGCUCAG**UUCAGCAGGAACAG	100.00
bta-miR-27a-3p	MIMAT0003532	U**UCACAGU**GGCUAAGUUCCG	hsa-miR-27a-3p	MIMAT0000084	U**UCACAGU**GGCUAAGUUCCGC	95.24
bta-miR-29a	MIMAT0003518	CU**AGCACCA**UCUGAAAUCGGUUA	hsa-miR-29a-3p	MIMAT0000086	U**AGCACCA**UCUGAAAUCGGUUA	95.45
bta-miR-324	MIMAT0009285	C**GCAUCCC**CUAGGGCAUUGGUGU	hsa-miR-324-5p	MIMAT0000761	C**GCAUCCC**CUAGGGCAUUGGUG	95.65
bta-miR-326	MIMAT0009286	C**CUCUGGG**CCCUUCCUCCAG	hsa-miR-326	MIMAT0000756	C**CUCUGGG**CCCUUCCUCCAG	100.00
bta-miR-345-3p	MIMAT0012535	**CCUGAA**CUAGGGGUCUGGAG	hsa-miR-345-3p	MIMAT0022698	G**CCCUGAA**CGAGGGGUCUGGAG	86.36
bta-miR-410	MIMAT0009311	A**AUAUAAC**ACAGAUGGCCUGU	hsa-miR-410-3p	MIMAT0002171	A**AUAUAAC**ACAGAUGGCCUGU	100.00
bta-miR-652	MIMAT0024578	A**AUGGCGC**CACUAGGGUUGUG	hsa-miR-652-3p	MIMAT0003322	A**AUGGCGC**CACUAGGGUUGUG	100.00
bta-miR-873	MIMAT0009377	G**CAGGAAC**UUGUGAGUCUCCU	hsa-miR-873-5p	MIMAT0004953	G**CAGGAAC**UUGUGAGUCUCCU	100.00
bta-miR-708	MIMAT0009367	A**AGGAGCU**UACAAUCUAGCUGGG	hsa-miR-708-5p	MIMAT0004926	A**AGGAGCU**UACAAUCUAGCUGGG	100.00
bta-miR-677	MIMAT0012003	C**UCACUGA**UGAGCAGCUUCUGAC	–	–	–	–

^1^For upstream analysis, only sequences with 90%-100% similarity and with preserved seed region were used. Seed regions are highlighted in bold in the sequence of each miRNA

**Fig. 5:**
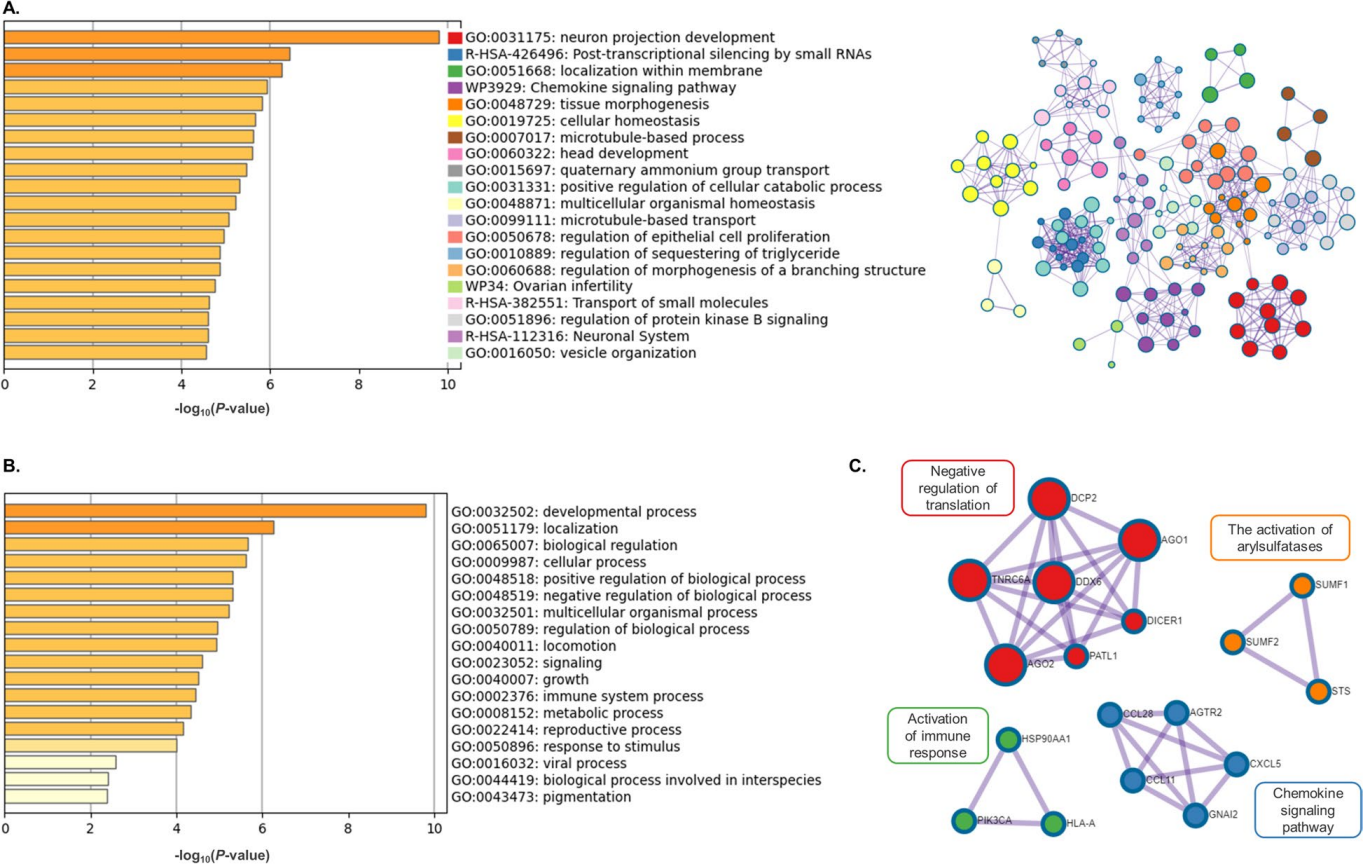
Interactions of miRNA up-regulated in OF-EVs and its modulated target genes. **A** Bar graph of enriched terms across input gene lists colored by *P*-values (left) and network of enriched terms colored by cluster ID (right). The size of each network node is proportional to the number of input genes. **B** The 20 top-level Gene Ontology biological processes. **C** Protein–protein interaction enrichment analysis. All the figures were generated using Metascape

**Fig. 6:**
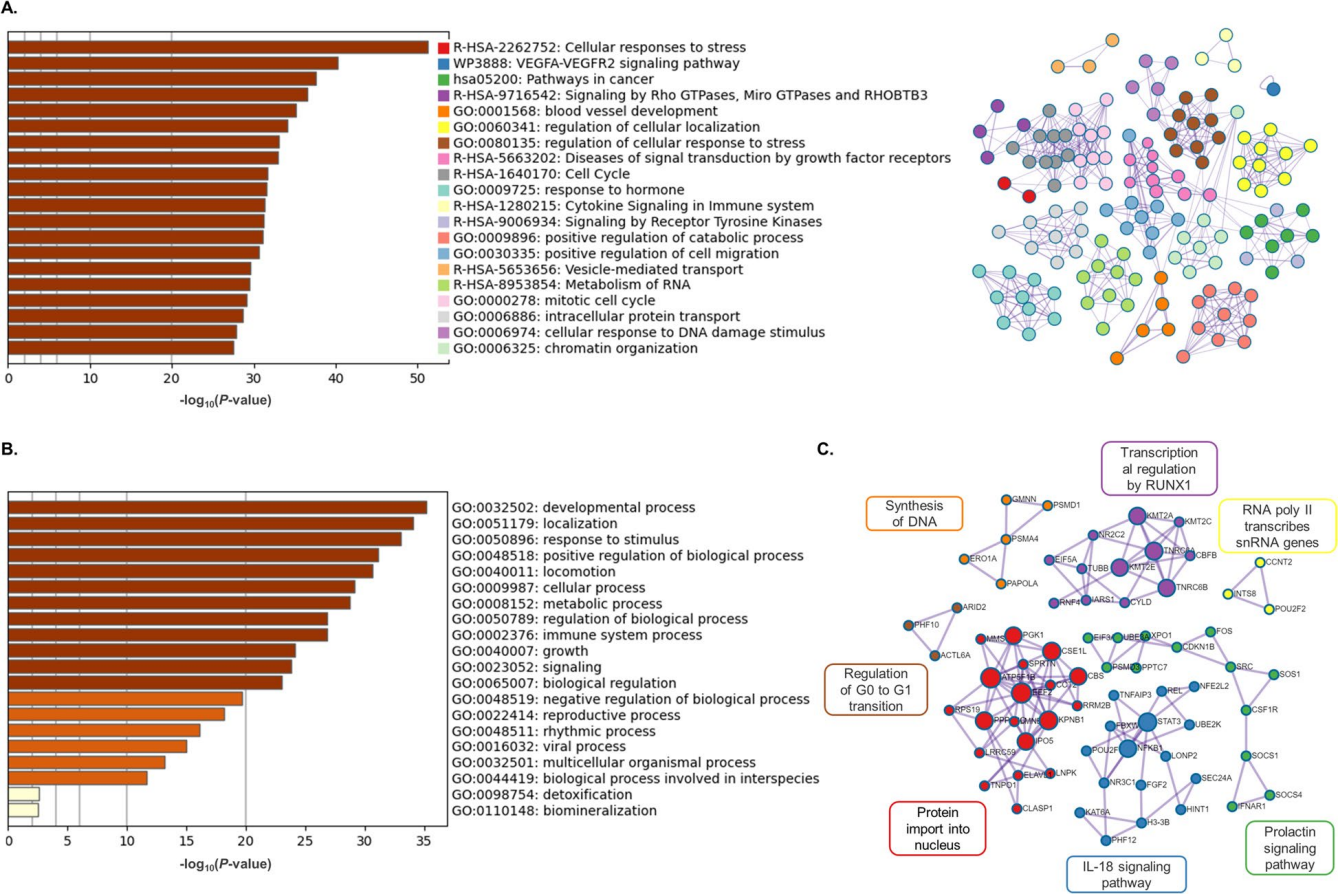
Interactions of miRNAs up-regulated in UF-EVs and their modulated target genes. **A** Bar graph of enriched terms across input gene lists colored by *P*-values (left) and network of enriched terms colored by cluster ID (right). The size of each network node is proportional to the number of input genes. **B** The 20 top-level Gene Ontology biological processes. **C** Protein–protein interaction enrichment analysis. All the figures were generated using Metascape

Metascape images represent functional enrichment analysis for genes modulated by miRNAs up-regulated in OF-EVs (miR-148b; Fig. 5) and miRNAs up-regulated in UF-EVs (miR-134-5p, miR-148b-3p, miR-151a-3p, miR-155-5p, miR-188-5p, miR-181b-5p, miR-181d-5p, miR-224-5p, miR-23b-3p, miR-24-3p, miR-27a-3p, miR-29a-3p, miR-324-5p, miR-326, miR-410-3p, miR-652-3p, miR-873-5p and miR-708-5p; Fig. 6). Figs. 5A and 6A represent the bar graph of 20 statistically enriched terms from the full cluster (left image) of miRNAs up-regulated in OF-EVs and miRNAs up-regulated in UF-EVs, respectively. Additionally, each one of the terms were converted into a network layout (right image). On the networks, the enriched terms are represented by a circle node, colored by cluster ID, where its size is proportional to the number of input genes. In both networks represented in Figs. 5A and 6A, it is possible to note the overlap and the interaction of genes between different clusters as there are connections among circle nodes.

Figures 5B and 6B represent the 20 top-level GO biological processes of miRNAs up-regulated in OF-EVs and miRNAs up-regulated in UF-EVs, respectively. miRNAs up-regulated in OF-EVs can modulate developmental processes. This analysis showed that miRNAs up-regulated in OF-EVs-mRNA interactions are enriched for biological pathways related with “immune process”, “metabolic process”, “cellular process” and “reproductive process”, similar to the miRWalk enrichment analysis. The miRNAs up-regulated in UF-EVs-mRNA interactions are enriched for biological pathways related with “cell cycle”, “immune process”, “metabolic process”, “cellular process” and “reproductive process”, also in congruence with the previous miRWalk enrichment analysis.

Figures 5C and 6C represent the protein–protein interaction enrichment analysis based on the miRNAs up-regulated in OF-EVs and miRNAs up-regulated in UF-EVs, respectively. miRNAs up-regulated in OF-EVs can modulate the activation of the immune response, while up-regulated in UF-EVs are related with synthesis of DNA, regulation of G0 to G1 transition, and prolactin signaling pathway. The 100 top-level GO biological processes and the complete list of all statistically enriched terms and related genes to miRNAs up-regulated in OF-EVs as well as to miRNAs up-regulated in UF-EVs are available as supplementary materials (Additional files 5 and 6).

### Lipid metabolism-related genes and differentially expressed miRNAs

As miRNA targeted genes to investigate, we have selected LMGs from our previous study, which showed that bovine embryos cultured in vitro in presence of EVs from OF and UF had reduced lipid contents and altered expression of some LMGs [21]. Selected LMGs are related with different processes of lipid metabolism, including lipid uptake, lipid transport, lipid accumulation, lipogenesis, and lipolysis (*LDLR*, *CD36*, *FABP3*, *PLIN2*, *PPARGC1B*, *ACACA*, *FASN*, *PNPLA2* and *LIPE*). Fatty acid synthesis genes also previously described as expressed by bovine embryos in another study were additionally analyzed (*ACSL3*, *ELOV5*, and *ELOV6*) [38].

**Fig. 7:**
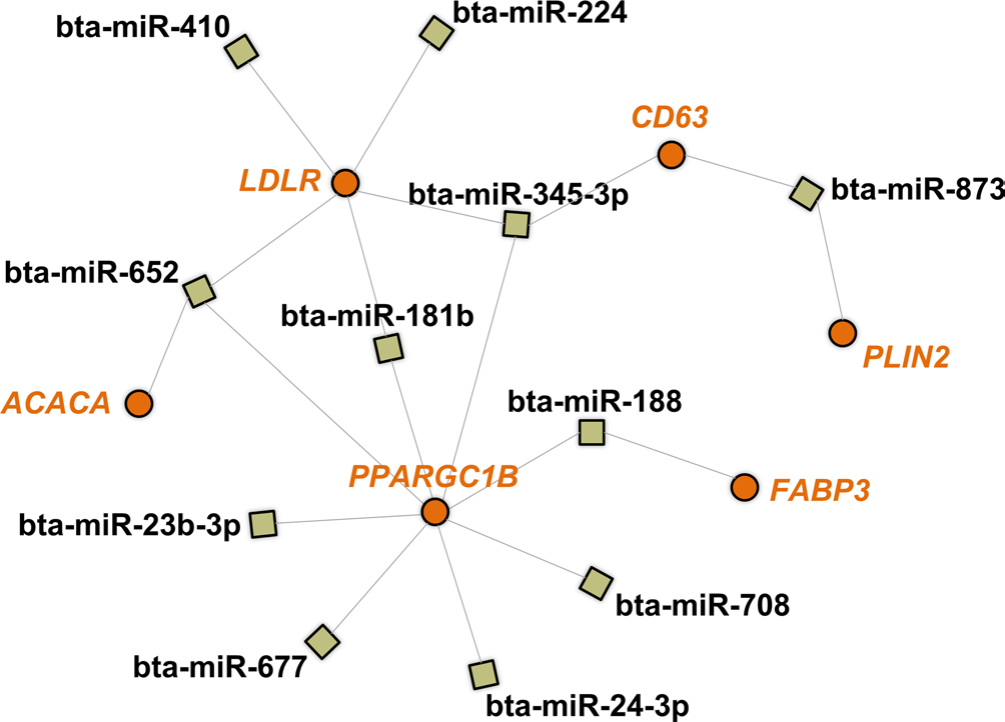
Network of lipid metabolism genes predicted to be modulated by miRNAs up-regulated in UF-EVs. Genes predicted to be modulated by miRNAs up-regulated in UF-EVs are represented in orange and miRNAs in black

**Fig. 8:**
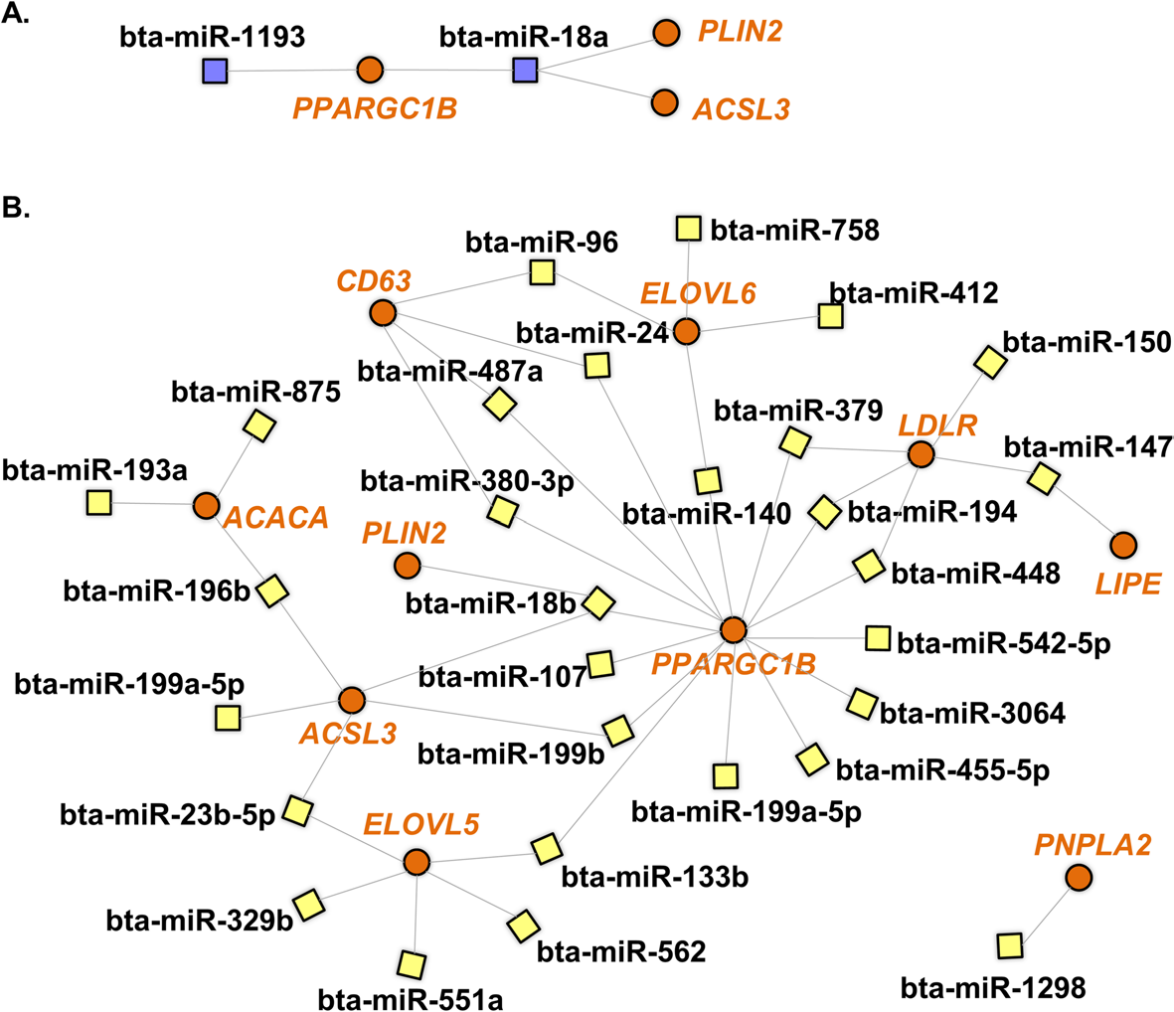
Network of lipid metabolism genes predicted to be modulated by exclusively detected miRNAs. **A** Genes (orange) predicted to be modulated by miRNAs exclusive to UF-EVs. **B** Genes (orange) predicted to be modulated by miRNAs exclusively in UF-EVs

In our first analysis, we used the miRWalk database to search for genes predicted as modulated by bovine miRNAs sequences. As seen in Fig. 7, none of the selected genes are predicted as targets of the miRNA up-regulated in OF-EVs, while 6 of them were predicted as targets of 11 out of the 19 miRNAs up-regulated in EVs from UF (*LDLR*, *CD36*, *FABP3*, *PPARGC1B*, *ACACA*, and *PLIN2*). As seen in Fig. 8A, 2 of 11 miRNAs exclusive to OF-EVs (bta-miR-1193 and bta-miR-18a) are predicted to modulate 3 LMGs (*PPARGC1B*, *PLIN2*, and *ACSL3*). As seen in Fig. 8B, 29 of 55 miRNAs exclusive to UF-EVs are predicted to modulate LMGs: *LDLR*, *CD36*, *PLIN2*, *PPARGC1B*, *ACACA*, *PNPLA2*, *LIPE*, *ACSL3*, *ELOV5* and *ELOV6*.

**Fig. 9:**
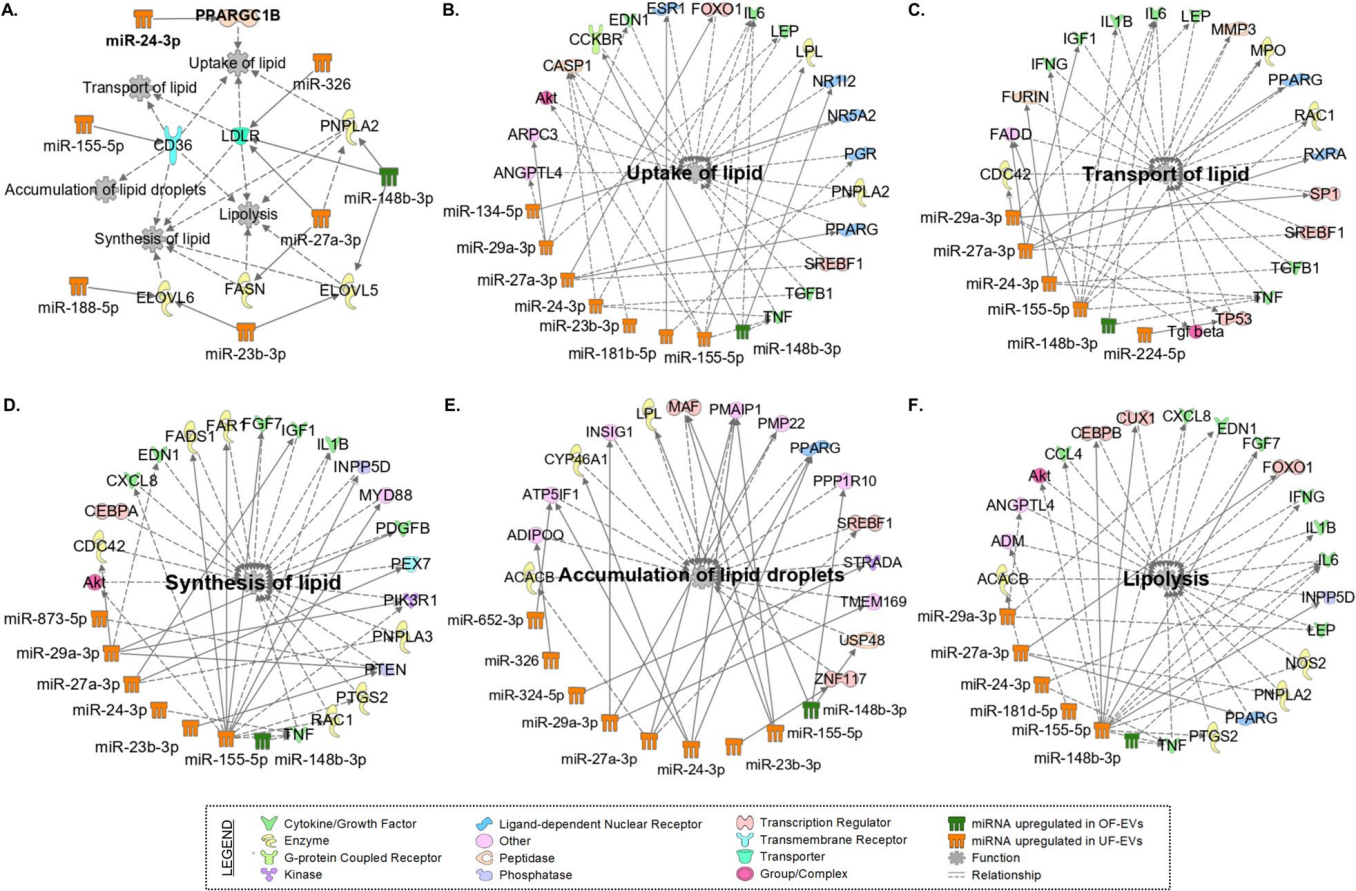
Network of lipid metabolism genes modulated by miRNAs differentially expressed between OF-EVs and UF-EVs. **A** Selected LMGs found to be modulated by differently expressed miRNAs (*PPARGC1B*, *CD36*, *LDLR*, *PNPLA2*, *ELOVL6*, *FASN*, and *ELOVL5*). **B** Networks of miRNAs and genes related with the uptake of lipids. **C** Networks of miRNAs and genes related with the transport of lipid. **D** Networks of miRNAs and genes related to the synthesis of lipids. **E** Networks of miRNAs and genes related with accumulation of lipid droplets. **F** Networks of miRNAs and genes related to lipolysis. Continuous lines indicate a direct relationship, while dashed lines indicate an indirect relationship. Networks were generated by QIAGEN Ingenuity Pathway Analysis software and only experimentally observed relationships were selected

In the context of our study on miRNAs, no validated targets have been identified in the bovine species. Therefore, these interactions were further investigated using the QIAGEN Ingenuity Pathway Analysis (IPA) software, wherein only experimentally observed relationships (rather than predicted bindings) were selected. Seven selected LMGs were found to be modulated by differentially expressed miRNAs: *PPARGC1B*, *CD36*, *LDLR*, *PNPLA2*, *ELOVL6*, *FASN*, and *ELOVL5* (Fig. 9A). Additionally, when using the IPA software to search for the keywords "uptake of lipid" (Fig. 9B), "transport of lipid" (Fig. 9C), "synthesis of lipid" (Fig. 9D), "accumulation of lipid droplets" (Fig. 9E), and "lipolysis" (Fig. 9D), it is indicated that miRNAs with distinct expression patterns in OF-EVs and UF-EVs possess the ability to regulate a multitude of genes beyond the selected LMGs.

## Discussion

Extracellular vesicles play an important role in embryo development, especially through their miRNA cargo [40]. Studies have shown that embryos cultured with OF-EVs and UF-EVs have reduced lipid contents, and their lipid metabolism genes were influenced by the presence of these vesicles [21]. Given that miRNAs are known to be important for bovine preimplantation embryo development, this study aimed to investigate deeper into the potential roles of miRNAs within EVs isolated from the oviductal and uterine regions of the female reproductive tract, which play a key role during the preimplantation stages of embryo development.

Our findings indicate that EVs derived from distinct parts of the female reproductive system (oviduct and uterus) during different stages of the estrous cycle (early and mid-luteal phases, respectively) exhibit variations in their miRNA content. These differences are concurrent with the progression of preimplantation development, specifically from the zygote until the 16-cell stage for oviduct-derived EVs and from the morula until blastocyst stage for uterus-derived EVs. Although it is important for future approaches to increase the number of samples and include pregnant animals to enhance our understanding of the regulation and crosstalk through miRNAs within EVs during pregnancy, the observed differences in miRNA content in our results using cyclic animals suggest the possibility of distinct regulatory effects on the embryo and its surrounding microenvironment. miRNAs present in these EVs may play a role in regulating LMGs and other crucial pathways involved in embryo development within the oviduct and uterus.

### The oviductal environment

The oviduct provides the ideal microenvironment for early embryonic development and participates on the modulation of the epigenetic landscape of the embryo [41, 42]. Different groups have already shown that EVs from the OF or from BOECs conditioned culture medium are internalized by bovine embryos and improve in vitro embryo development and quality [14, 19, 21]. These results could be due to the delivery of miRNAs within the EVs that through epigenetic modulation favors the embryonic development in vitro. The only miRNA (bta-miR-148b) with higher expression level in OF-EVs is reported to affect fertilization and embryo development by modulating the expression of PTEN (phosphatase with tensin homology deleted in chromosome 10) [43]. PTEN is essential for embryonic development [44], and in mice, its deletion causes embryonic lethality [45], emphasizing the importance of PTEN regulation. Furthermore, bioinformatics analysis suggests a connection between this miRNA and the TGF-β biological pathway, associated with blastocyst formation and embryo lineage segregation. Indeed, recent data from our group demonstrates that supplementing in vitro culture with bta-miR-148b mimics enhances embryo quality through the modulation of the TGF-β signaling pathway [46]. This modulation leads to alterations in gene transcription related to cellular differentiation and proliferation, suggesting the oviduct’s role in promoting embryo quality and development.

Additionally, bta-miR-148b is predicted to modulate 17 signaling pathways (KEEG). Among the identified pathways, AMPK (*P* = 0.0144), Ras (*P* = 0.0217), and Wnt (*P* = 0.0235) exhibit the most statistically significant associations. The 11 miRNAs exclusive to OF-EVs are predicted to modulate 48 signaling pathways, and among the identified pathways with the lowest *P*-value are Ras (*P* = 0.0015) and ErbB (*P* = 0.002). The canonical Wnt signaling is recognized as a crucial mechanism involved in cell proliferation, pluripotency maintenance, differentiation, and migration [47]. Data from the mouse shows that the Wnt system is present and active as early as the two-cell stage [48] and in bovine embryos, the WNT signaling system plays important roles in directing developmental processes, including maintenance of pluripotency, and its activation of the canonical pathway can inhibit embryonic development [49]. Additionally, seven pathways were identified as modulated only by miRNAs in OF-EVs, and from these, 3 are related to cell metabolism, including 2 pathways related with lipid metabolism and degradation, which will be discussed later.

### The uterine environment and embryo differentiation

The uterine horn receives the bovine embryo (> 16 cell stage/early morula) on d 4/5 after fertilization, and its endometrial epithelium is the contact point between the maternal tissue and the developing embryo [11]. Our study demonstrated that during the mid-luteal phase of the estrous cycle, period where the embryo will enter the uterus, the 19 miRNAs up-regulated in UF-EVs are predicted to modulate 101 signaling pathways, and the 59 exclusives to UF-EVs are predicted to modulate 78 signaling pathways. Previous studies have shown that miRNAs from the endometrium are uptaken and may modify the transcriptome of the preimplantation embryo [50]. During the preimplantation embryo development, blastocyst formation is an important milestone and miRNAs have been related with its early lineage segregation through the modulation of pluripotency and cell differentiation [51]. Interestingly, bta-miR-155, up-regulated in UF-EVs, is mainly expressed in the bovine inner cell mass (ICM) and is involved in cell motility, morphogenesis, and apoptosis [52]. Additionally, its overexpression has a positive impact on mice embryo development [53]. Furthermore, miR-24-3p, also up-regulated in UF-EVs, regulates the differentiation of germ-layer in early mice embryos through the downregulation of pluripotency markers such as *Oct4*, *Nanog*, *KLF4*, and *c-Myc* [54]*.* Bioinformatic analysis also suggests the potential role of miRNAs within UF-EVs on early embryonic development.

The KEEG functional enrichment showed that the Hippo signaling pathway is among 15 pathways highly modulated by miRNAs exclusive to UF-EVs. Hippo is one of the main regulators of trophectoderm and ICM differentiation in mice [55] and in porcine embryos [56]. Although the localization of Hippo components in bovine is significantly different from mouse embryogenesis, in bovine it is also associated with cell fate specification and lineage segregation during the formation of a blastocyst [57]. Additionally, in mouse blastocyst, the combination of the Hippo pathway with NOTCH pathway modulates the specification of the trophectoderm [58]. Of note, NOTCH signaling pathway is only modulated by miRNAs exclusive to UF-EVs, and its proper modulation is related with cell proliferation in bovine early embryos [59]. Therefore, our results indicate that UF-EVs are potentially carriers for the delivery of maternal miRNAs that through modulation of signaling pathways can regulate pluripotency, differentiation, and early lineage segregation.

### Uterine receptivity and implantation

To ensure proper embryo development, an optimal uterine environment is also indispensable for successful embryo reception. miRNA identified in OF- and UF-EVs, such as bta-let-7a, bta-let-7b, bta-miR-151, bta-miR-181, bta-miR-29a, and bta-miR-494 have been associated with implantation in sheep [60], mice [61], and humans [62]. Additionally, from the 20 differentially expressed miRNAs, nearly all of them (19) were in higher expression levels in UF-EVs, suggesting that these miRNAs could be involved with the embryonic arrival to the uterus. Notably, miR-148b, the only miRNA with lower expression levels in UF-EVs, participates in the regulation of cell progression and high concentrations of this miRNA were found in the endometrium of women with failed implantation [63]. This miRNA has also been related with the attenuation of inflammatory response in bovine endometrial epithelial cells (BEECs) [64]. This observation suggests that the reduction of bta-miR-148b in the uterus compared to the oviduct may be associated with a favorable implantation process, but more studies are needed to investigate this hypothesis.

Furthermore, miR-324, miR-224, miR-652, up-regulated in UF-EVs, have been associated with uterine receptivity and implantation in humans and mice. In humans, miR-324 is highly associated with *FOXO1* and it is a candidate for identifying the receptivity of the endometrium through endometrial fluid biopsy and the up-regulation of miR-224 in the endometrial fluid is associated with the receptive phase [65]. In vitro, the upregulation of miR-652, regulates the viability, proliferation, and invasion of mice trophoblast cells [66]. Taken together, the miRNAs up-regulated in UF-EVs could be related with the modulation of the endometrial cells to support future implantation processes. And of greater significance, miR-324 and miR-224 serve as non-invasive fertility biomarkers in humans. Exploring these miRNAs within EVs in bovine reproductive processes could offer valuable insights for fertility assessment before embryo transfer. Nonetheless, further studies are required to examine this hypothesis.

### Immune modulation by miRNAs within EVs

Since the embryo is a semi-allograft challenge to the maternal immune system, both oviduct and uterus must be correctly programmed to allow embryo development and at the same time protect this environment against pathogens [67]. In this way, miRNAs and EVs serve as key regulators in intercellular communication and mediation of immune responses and inflammation including during pregnancy [68]. It is well known that bovine embryos express MHC-I transcripts and thus may be recognized as foreign already in the oviduct [69]. The downregulation of MHC-I is a common immune evasion mechanism of pathogens and bta-miR-148b, up-regulated in OF-EVs in our results, is reported to target and downregulate MHC-I in BEECs [64]. We suggest that although this miRNA was up-regulated in OF-EVs, it is acting in both organs and may participate in the fine tuning of the embryo MHC-I. Additionally, bta-miR-29a, up-regulated in UF-EVs, has already been identified as up-regulated in UF-EVs of cows on d 7 of pregnancy and negatively regulates the uterine innate immunity, protecting the pre-hatching blastocysts [70].

In bovine endometrial cells, progesterone (P4) acts by downregulating the expression of proinflammatory genes, inhibiting the activation of MAPK and NF-κB pathways [71]. Interestingly, among the 15 pathways modulated by miRNAs with lower *P*-values, MAPK is exclusively up-regulated in UF-EVs, suggesting its critical role in the uterine environment. Additionally, bta-miR-24-3p, up-regulated in UF-EVs, leads to the control of the NF-κB signaling pathway, involved in the pro-inflammatory response [72]. Although Talukder et al. [73] and Maillo et al. [10] propose that in the oviduct, the embryo’s presence stimulates the suppression of key factors of the NF-κB pathway, such as NFkB2 and NFkBIA, resulting in an anti-inflammatory response [10, 73], our results indicate that NF-κB is predicted to be modulated only by UF-EVs miRNAs. Therefore, in the oviduct, the modulation of the NF-κB signaling pathway may be more strongly influenced by the presence of the embryo. On the other hand, even in the absence of the embryo, the miRNAs within UF-EVs might act in a paracrine manner to suppress NF-κB signaling and create a local environment that favors embryo development in the uterus.

However, it is worth considering that the presence of pre-hatching embryos and their communication with the maternal immune system are crucial for successful pregnancy [74]. One limitation of our work is that the OF-EVs and UF-EVs were collected from cyclic animals. Despite the absence of the embryo and its interferon tau (IFN-T) secretion and immune regulation, cows were under P4 regulation, which is an immunosuppressive molecule [75]. Additionally, the oviductal and uterine environment must be already prepared with the ideal conditions for embryonic development before embryo arrives. Indeed, Metascape analysis suggests that the miRNAs identified in OF-EVs could modulate the immune system process (GO) and the activation of immune response (protein–protein interaction enrichment analysis). Similarly, UF-EVs also modulates immune system process (GO), as well as cytokine signaling in immune system (GO) and IL-18 signaling pathway (protein–protein interaction enrichment analysis). Taken together, our results suggest that the miRNAs found both in OF-EVs and UF-EVs are potentially involved in the immune balance that will induce embryo-maternal immune tolerance, ensuring proper embryo development and successful pregnancy.

### Modulation of LMGs

Based on our previous investigation, embryos cultured with EVs from OF and UF exhibited lower lipid contents and altered expression of LMG [21]. Here we identified miRNAs in these EVs and investigated their impact on lipid metabolism pathways. Bioinformatical analysis showed that both OF- and UF-EVs modulate these pathways, with lipid metabolism and lipid degradation being exclusively modulated by OF-EVs miRNAs. Lipids serve as a crucial energy reservoir for early embryonic development [76]. In vitro-produced (IVP) embryos have higher lipid content compared to in vivo-derived (IVD) embryos and disruptions in lipid metabolism during in vitro culture led to excessive lipid droplet formation, negatively impacting cryotolerance and mitochondrial function [77]. Therefore, proper lipid metabolism and degradation, especially in the in vitro environment, seems to be crucial for proper embryo development and our results suggest that this modulation could be significantly influenced by miRNAs within EVs.

To further assess the association of differentially expressed miRNAs with lipid metabolism, we also focused on LMG known to be expressed in bovine embryos, which could potentially be regulated by these miRNAs. Notably, among the 19 up-regulated miRNAs in UF-EVs, 11 were predicted to control specific LMGs: *LDLR*, *CD36*, *FABP3*, *PPARGC1B*, *ACACA*, and *PLIN2*. Similarly, we conducted a similar analysis focusing on miRNAs exclusively found in OF- and UF-EVs. Among the 11 miRNAs exclusive to OF-EVs, 2 (bta-miR-1193 and bta-miR-18a) were predicted to be associated with lipid metabolism, regulating 3 genes (*PPARGC1B*, *PLIN2*, and *ACSL3*). For the 51 miRNAs exclusive to the UF-EVs, 28 of them were predicted to modulate lipid metabolism, controlling 10 of the 12 genes studied (only *FAPBP3* and *FASN* were not predicted). These genes are involved in various aspects of lipid metabolism, including uptake and transport (*LDLR*, *CD36*, and *FABP3*) [78–80], lipogenesis (*PPARGC1B* and *ACACA*) [81, 82], and lipid accumulation (*PLIN2*) [83]. Thus, different miRNAs present in OF- and UF-EVs, have the potential to regulate the expression of key genes involved in lipid metabolism in bovine embryos.

In a previous study, Leal et al. [21], observed that when cultured with OF- and UF-EVs, the genes *LDLR*, *FASN*, *PNPLA2*, and *PLIN2* were down-regulated, and embryos showed lower lipid droplets (LD) content. *LDLR* is predicted to be modulated by 10 miRNAs present in UF-EVs, and there is a validated interaction between this gene and miR-326 and miR-27a-3p, which are up-regulated in UF-EVs. *LDLR* promotes the uptake of low-density lipoprotein (LDL), which is the major carrier of cholesterol and as shown by Sato et al. [84], *LDLR* may play an essential role in the uptake of exogenous LDL into mice blastocysts [84]. Therefore, we suggest that through the downregulation of this receptor by miRNAs within EVs, there could also be a reduction in lipid accumulation. *PLIN2* is predicted to be modulated only by miRNAs present in UF-EVs (bta-miR-873 and bta-miR-18a). This gene is known to inhibit lipid degradation, thereby promoting lipid accumulation [85]. Additionally, previous reports suggest that *PLIN2* may contribute to the preservation of lipid reserves during early embryo development in cattle [86]. Therefore, we hypothesize that these miRNAs, which reduce the expression of *PLIN2* genes, could also downregulate the accumulation of LD. Further studies utilizing specific miRNAs, such as miR-326, miR-27a-3p, bta-miR-873, or bta-miR-18a, during in vitro culture could serve as a valuable approach to reduce lipid content, consequently enhancing post-thaw viability.

Additionally, an interesting observation from our analysis was that the *PPARCG1B* gene exhibited the highest number of miRNAs predicted to regulate its expression in both OF- and UF-EVs. *PPARGC1B* functions as a transcriptional coactivator for peroxisome proliferator-activated receptor gamma (PPARG) and participates in the regulation of fatty acid metabolism [81]. This transcript has been previously identified in bovine embryos [87], and studied in association with lipid metabolism [88]. Furthermore, the IPA software identifies PPARCG1B as a validated target of miR-24-3p. Notably, our findings revealed downregulation of PPARGC1B in blastocysts treated with OF- and UF-EVs [21], coinciding with the upregulation of miR-24-3p in UF-EVs. These results suggest that the miRNAs present in EVs, particularly miR-24-3p, may play a crucial role in modulating lipid metabolism and influencing the expression of PPARGC1B during early embryo development. This hypothesis remains to be confirmed and the expression and function of *PPARGC1B* in bovine embryos would be interesting to be further investigated.

## Conclusion

In conclusion, our study shows that OF- and UF-EVs have different miRNA profiles. The functional enrichment analysis revealed that both OF- and UF-EVs miRNAs could influence pathways related to cell proliferation, differentiation, and the immune system, which are crucial for reproductive tract and embryo development. Furthermore, despite the limitation of a relatively low sample size, our study indicates that miRNAs within both OF- and UF-EVs are potentially involved in maternal-embryonic communication within the oviduct and uterus, promoting early embryo development and contributing to a successful pregnancy. Nevertheless, we highlighted the potential role of miRNAs in EVs, particularly those derived from the uterus, in regulating embryo lipid metabolism, with PPARGC1B emerging as an interesting target to understand the impact of miRNAs on lipid accumulation. Further studies will elucidate the abundance, distribution, and form of miRNAs within EVs in maternal environments, and functional experiments are needed to validate the function of specific miRNA following uptake by the embryo. Analyzing miRNA expression in these EVs provides valuable insights into maternal-embryonic communication during different stages of early development, with significant implications for understanding embryo development and maternal support through EVs during pregnancy.

### Supplementary Information

**Additional file 1.** Chromosome location and family precursor structure and sequence conservation of differentially expressed miRNAs.

**Additional file 2:** **A**. All enriched signaling pathways and number of genes (hits) predicted to be regulated by miRNAs exclusive in OF-EVs. **B**. All enriched signaling pathways and number of genes (hits) predicted to be regulated by miRNAs exclusive in UF-EVs. **C**. All enriched signaling pathways and number of genes (hits) predicted to be regulated by the miRNA up regulated in OF-EVs. **D**. All enriched signaling pathways and number of genes (hits) predicted to be regulated by miRNAs up regulated in UF-EVs.

**Additional file 3.** miRNA-mRNA target interactions by miRNAs up-regulated in OF-EVs and UF-EVs.

**Additional file 4.** miRNA-mRNA target interactions by miRNAs exclusives to OF-EVs and UF-EVs.

**Additional file 5.** 100 top-level gene ontology biological processes.

**Additional file 6: A**. Complete list of all statistically enriched terms and related genes to miRNAs upregulated in OF-EVs. **B**. Complete list of all statistically enriched terms and related genes to miRNAs upregulated in UF-EVs.

## Data Availability

The raw data supporting the conclusion of this article will be made available by the authors, without undue reservation, to any qualified researcher. In addition, all generated, or analyses data derived from this study are included in this published article and its Supplementary data files.

## Abbreviations

BEECs: Bovine endometrial epithelial cells
BOECs: Bovine oviduct epithelial cells
Ct: Cycle threshold
EVs: Extracellular vesicles
GO: Gene Ontology
IFN-T: Interferon tau
IVC: In vitro culture
IVD: In vivo-derived
KEGG: Kyoto Encyclopedia of Genes and Genomes
LD: Lipid droplets
LDL: Low-density lipoprotein
LMG: Lipid metabolism-related gene
miRNAs: MicroRNAs
OF: Oviductal fluid
P4: Progesterone
PBS-: Calcium and magnesium-free phosphate-buffered saline
qRT-PCR: Quantitative real-time PCR
SEM: Standard error of the mean
UF: Uterine fluid
