# Correction: Extracellular vesicles-coupled miRNAs from oviduct and uterus modulate signaling pathways related to lipid metabolism and bovine early embryo development

**DOI:** 10.1186/s40104-024-01035-2

**Published:** 2024-04-24

**Authors:** Rosane Mazzarella, Karina Cañón-Beltrán, Yulia N. Cajas, Meriem Hamdi, Encina M. González, Juliano C. da Silveira, Claudia L. V. Leal, D. Rizos

**Affiliations:** 1 Department of Animal Reproduction, INIA-CSIC, Madrid, Spain; 2 Department of Biochemistry and Molecular Biology, Veterinary Faculty, Complutense University of Madrid (UCM), Madrid, Spain; 3 Department Agrarian Production, Technical University of Madrid, UPM, Madrid, Spain; 4 Departamento de Ciencias Biológicas, Universidad Técnica Particular de Loja, UTPL, Loja, Ecuador; 5 Department of Anatomy and Embryology, FV-UCM, Madrid, Spain; 6 Department of Veterinary Medicine, FZEA-USP, Pirassununga, Brazil

**Correction** **:** **J Animal Sci Biotechnol 15, 51 (2024)**

**https://doi.org/10.1186/s40104-024-01008-5**

Following publication of the original article [1], the authors reported that the last two sentences were mistakenly placed at the end of the Results section in the Abstract which should have been placed at the beginning of this section.

The original Results section in the Abstract was:

From the 20 differentially expressed miRNAs, 19 up-regulated in UF-EVs (bta-miR-134, bta-miR-151-3p, bta-miR-155, bta-miR-188, bta-miR-181b, bta-miR-181d, bta-miR-224, bta-miR-23b-3p, bta-miR-24-3p, bta-miR-27a-3p, bta-miR-29a, bta-miR-324, bta-miR-326, bta-miR-345-3p, bta-miR-410, bta-miR-652, bta-miR-677, bta-miR-873 and bta-miR-708) and one (bta-miR-148b) in OF-EVs. These miRNAs were predicted to modulate several pathways such as Wnt, Hippo, MAPK, and lipid metabolism and degradation. Differences in miRNAs found in OF-EVs from the early luteal phase and UF-EVs from mid-luteal phase may reflect different environments to meet the changing needs of the embryo. Additionally, miRNAs may be involved, particularly in the uterus, in the regulation of embryo lipid metabolism, immune system, and implantation. This study evaluated miRNA cargo in OF-EVs from the early luteal phase and UF-EVs from the mid-luteal phase, coinciding with embryo transit within oviduct and uterus in vivo, and its possible influence on LMGs and signaling pathways crucial for early embryo development. A total of 333 miRNAs were detected, with 11 exclusive to OF, 59 to UF, and 263 were common between both groups.

The correct Results section in the Abstract should read:

This study evaluated miRNA cargo in OF-EVs from the early luteal phase and UF-EVs from the mid-luteal phase, coinciding with embryo transit within oviduct and uterus in vivo, and its possible influence on LMGs and signaling pathways crucial for early embryo development. A total of 333 miRNAs were detected, with 11 exclusive to OF, 59 to UF, and 263 were common between both groups. From the 20 differentially expressed miRNAs, 19 up-regulated in UF-EVs (bta-miR-134, bta-miR-151-3p, bta-miR-155, bta-miR-188, bta-miR-181b, bta-miR-181d, bta-miR-224, bta-miR-23b-3p, bta-miR-24-3p, bta-miR-27a-3p, bta-miR-29a, bta-miR-324, bta-miR-326, bta-miR-345-3p, bta-miR-410, bta-miR-652, bta-miR-677, bta-miR-873 and bta-miR-708) and one (bta-miR-148b) in OF-EVs. These miRNAs were predicted to modulate several pathways such as Wnt, Hippo, MAPK, and lipid metabolism and degradation. Differences in miRNAs found in OF-EVs from the early luteal phase and UF-EVs from mid-luteal phase may reflect different environments to meet the changing needs of the embryo. Additionally, miRNAs may be involved, particularly in the uterus, in the regulation of embryo lipid metabolism, immune system, and implantation.

The original article [1] has been updated.

## References

[1] Mazzarella R, Cañón-Beltrán K, Cajas YN (2024). Extracellular vesicles-coupled miRNAs from oviduct and uterus modulate signaling pathways related to lipid metabolism and bovine early embryo development. J Animal Sci Biotechnol.

