# Supplementary material for: Extracellular vesicles-coupled miRNAs from oviduct and uterus modulate signaling pathways related to lipid metabolism and bovine early embryo development
Source: J Anim Sci Biotechnol. 2024 Apr 4;15:51. doi: 10.1186/s40104-024-01008-5 (PMC10993494; doi:10.1186/s40104-024-01008-5)

Additional file 1. Chromosome location and family precursor structure and sequence conservation of differentially expressed miRNAs. One miRNA was upregulated in OF-EVs: (A) bta-miR-148b. Nineteen miRNAs up-regulated in OF-EVs: (B) bta-miR-134, (C) bta-miR-151-3p, (D) bta-miR-155, (E) bta-miR-188, (F) bta-miR-181b, (G) bta-miR-181d, (H) bta-miR-224, (I) bta-miR-23b-3p, (J) bta-miR-24-3p, (K) bta-miR-27a-3p, (L) bta-miR-29a, (M) bta-miR-324, (N) bta-miR-326, (O) bta-miR-345-3p, (P) bta-miR-410, (Q) bta-miR-652, (R) bta-miR-677, (S) bta-miR-873 and (T) bta-miR-708. Gene locations were accessed through RNAcentral v20 (Sweeney et al., 2021) and miRNAs family precursor structure and sequence conservation through Rfam 14.8 database and R2DT 1.2 software provided by Rfam (Kalvari et al., 2021). Nucleotide sequence: A in green, T in red, C in blue and G in yellow. Precursor structure: black structures. Sequence conservation: color graduation in red indicates nucleotides highly conserved and blue poorly conserved across species.

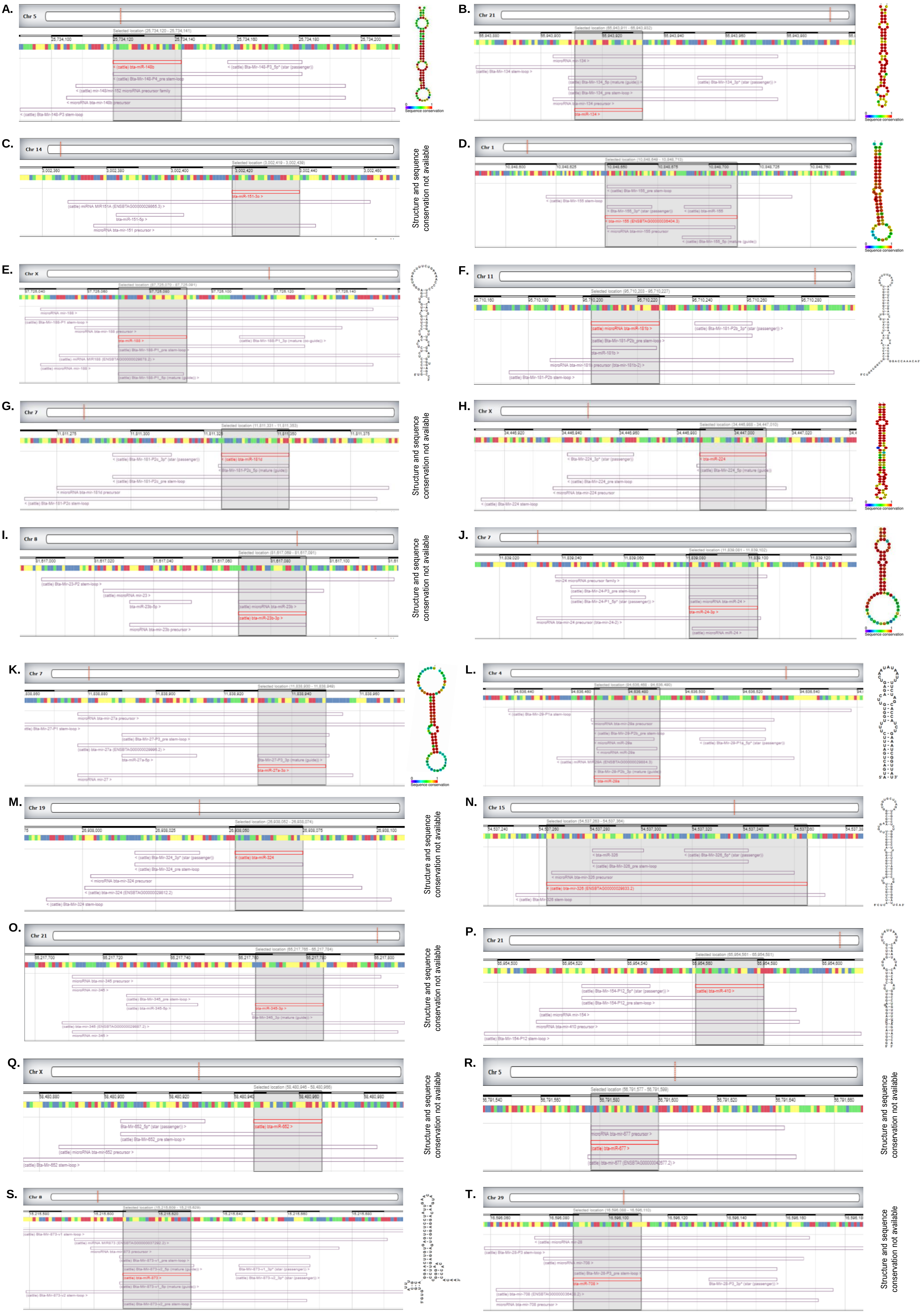

Supplement: Supplementary file 1 — Additional file 1. Chromosome location and family precursor structure and sequence conservation of differentially expressed miRNAs. [file 40104_2024_1008_MOESM1_ESM.pdf]
