# Supplementary material for: Extracellular vesicles-coupled miRNAs from oviduct and uterus modulate signaling pathways related to lipid metabolism and bovine early embryo development
Source: J Anim Sci Biotechnol. 2024 Apr 4;15:51. doi: 10.1186/s40104-024-01008-5 (PMC10993494; doi:10.1186/s40104-024-01008-5)

Additional file 5. 100 top-level gene ontology biological processes. (A) Biological processes related to miRNAs up-regulated in OF-EVs. (B) Biological processes related to miRNAs up-regulated in UF-EVs.

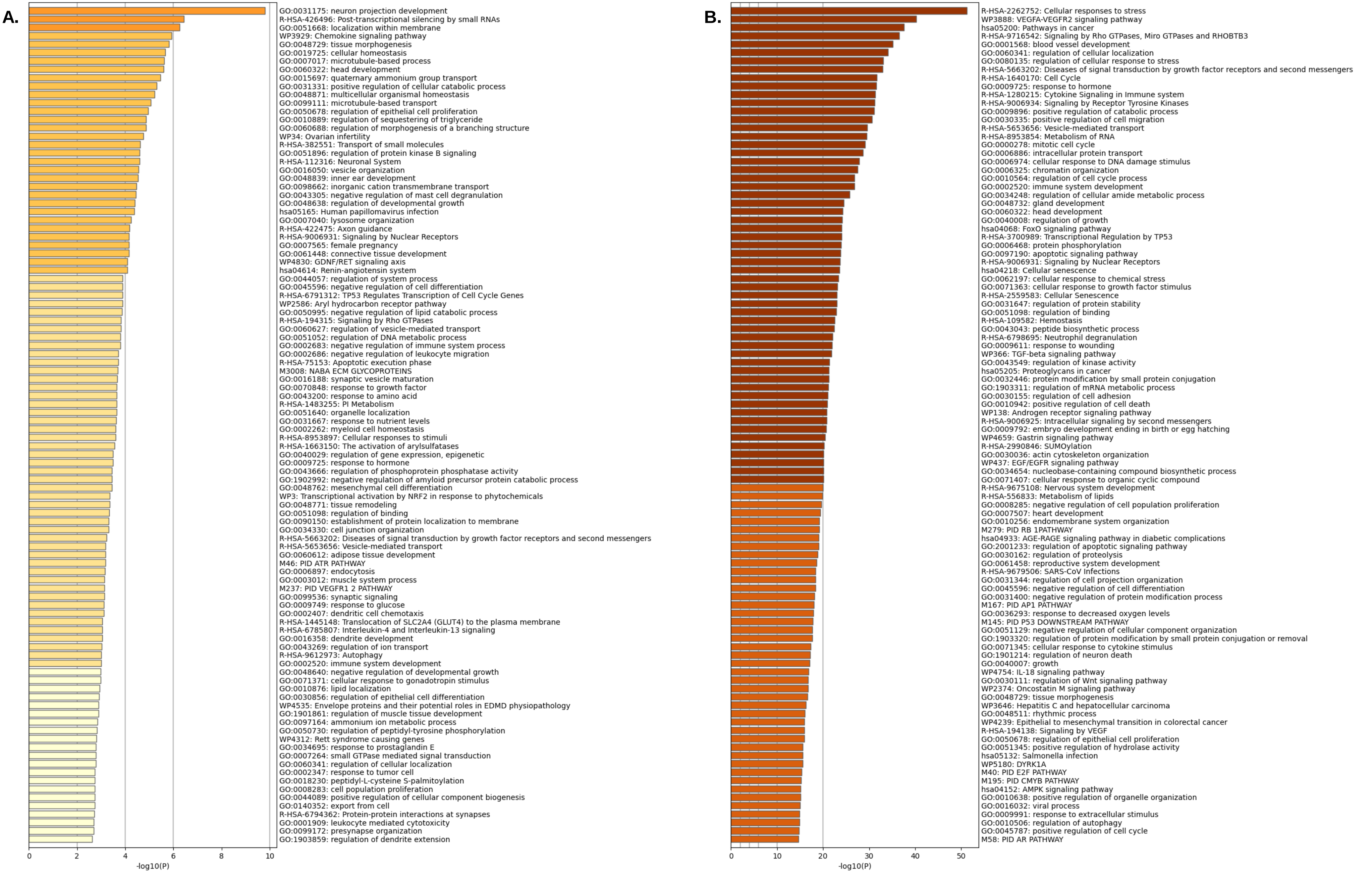

Supplement: Supplementary file 5 — Additional file 5. 100 top-level gene ontology biological processes. [file 40104_2024_1008_MOESM5_ESM.pdf]
